# *Desert hedgehog *is a mammal-specific gene expressed during testicular and ovarian development in a marsupial

**DOI:** 10.1186/1471-213X-11-72

**Published:** 2011-12-01

**Authors:** William A O'Hara, Walid J Azar, Richard R Behringer, Marilyn B Renfree, Andrew J Pask

**Affiliations:** 1 Department of Molecular and Cellular Biology, The University of Connecticut, Storrs CT 06269, USA; 2 Department of Zoology, The University of Melbourne, Victoria 3010, Australia; 3 Department of Genetics, The University of Texas M. D. Anderson Cancer Center, Houston, TX 77030, USA; 4 The Australian Research Council Centre of Excellence in Kangaroo Genomics, Australia

**Keywords:** Sex determination, sexual differentiation, gene expression, marsupial, *Macropus eugenii*.

## Abstract

**Background:**

*Desert hedgehog *(*DHH*) belongs to the hedgehog gene family that act as secreted intercellular signal transducers. DHH is an essential morphogen for normal testicular development and function in both mice and humans but is not present in the avian lineage. Like other hedgehog proteins, DHH signals through the patched (PTCH) receptors 1 and 2. Here we examine the expression and protein distribution of DHH, PTCH1 and PTCH2 in the developing testes of a marsupial mammal (the tammar wallaby) to determine whether DHH signalling is a conserved factor in gonadal development in all therian mammals.

**Results:**

*DHH*, *PTCH1 *and *PTCH2 *were present in the marsupial genome and highly conserved with their eutherian orthologues. Phylogenetic analyses indicate that *DHH *has recently evolved and is a mammal-specific hedgehog orthologue. The marsupial *PTCH2 *receptor had an additional exon (exon 21a) not annotated in eutherian PTCH2 proteins. Interestingly we found evidence of this exon in humans and show that its translation would result in a truncated protein with functions similar to PTCH1. We also show that *DHH *expression was not restricted to the testes during gonadal development (as in mice), but was also expressed in the developing ovary. Expression of *DHH*, *PTCH1 *and *PTCH2 *in the adult tammar testis and ovary was consistent with findings in the adult mouse.

**Conclusions:**

These data suggest that there is a highly conserved role for DHH signalling in the differentiation and function of the mammalian testis and that DHH may be necessary for marsupial ovarian development. The receptors PTCH1 and PTCH2 are highly conserved mediators of hedgehog signalling in both the developing and adult marsupial gonads. Together these findings indicate DHH is an essential therian mammal-specific morphogen in gonadal development and gametogenesis.

## Background

Desert hedgehog (*DHH*) is a member of the hedgehog gene family which act as secreted intercellular signal transducers [1]. Hedgehog was first identified as a segment polarity gene in *Drosophila *and has since been identified as a key regulator of pattern formation in embryonic and adult development in many vertebrate and invertebrate species. In addition, the *hedgehog *gene has undergone duplications in both invertebrates and vertebrates to produce a number of orthologues [2-6]. Mammals have three hedgehog orthologues, Sonic (*Shh*), Indian (*Ihh*), and desert (*Dhh*) hedgehog [3,4,7-9]. All three share a striking homology with the *Drosophila *orthologue [10]. Each mammalian orthologue has a unique, largely non-overlapping expression pattern, except in the gut where *Ihh *and *Shh *are co-expressed, and in the adult ovaries where *Ihh *and *Dhh *are co-expressed [11,12]. Shh has an essential role in early fetal development, and is required for correct formation of the limbs, phallus, somites and neural tube [8-10]. Ihh has a more restricted developmental role, and is essential for chondrocyte development [13]. Dhh is essential for the maintenance of the male germ line and spermatogenesis [14]. *Dhh *is also expressed in Schwann cells, and appears to play a role in nerve sheath formation [15,16].

Hedgehog actions are mediated at the cell surface by a multi-component receptor complex comprising the patched (PTCH) receptors and smoothened (SMO) protein [17]. Both proteins have orthologues in *Drosophila *that are also involved in hedgehog signal transduction and pattern formation. Initially, all three hedgehog protein functions were thought to be mediated through the PTCH1 receptor in mammals. However, a second orthologue was identified, *PTCH2*, which shares significant sequence homology with *PTCH1*. PTCH1 and PTCH2 both bind all hedgehog family members with similar affinities, and each forms a complex with SMO. However, the expression patterns of *PTCH1 *and *PTCH2 *do not entirely overlap, suggesting some degree of functional specialization [18]. While *PTCH1 *is widely expressed throughout the mouse embryo, *PTCH2 *is most predominant in the skin and in testis [18].

In the mouse, *Dhh *is expressed in the presumptive testis from E11.5 through to adult stages. *Dhh *was initially thought to be specifically expressed in the testis in the pre-Sertoli cells and so it was suggested it may be directly modulated by Sry [1,10,19] or its downstream partner Sox9 [20-22]. *Dhh*-null male mice are sex reversed [21,23], their gonads are small and devoid of sperm and the mice develop as phenotypic females due to a lack of male steroid hormone production [20,21]. Leydig cell numbers are dramatically decreased in the *Dhh *null gonad, but are not entirely absent [24]. Dhh appears to be an important regulator of Leydig cell development since its over-expression in the somatic cells can induce Leydig cell development [25]. In contrast to the *Dhh*-null males, female mice lacking the *Dhh *gene develop normally and are fertile [1]. While *Dhh *does not appear to be important for ovarian development, *Dhh *mRNA has been detected in the granulosa cells of preantral and antral follicles suggesting it can be activated in an SRY-independent manner [1,12,24-26]. In humans, mutations in DHH cause a range of phenotypes in 46, XY male patients from mild [27] to complete gonadal dysgenesis, including bilateral streak gonads, normally developed Müllerian ducts, and female external genitalia [28].

The majority of genes involved in testicular differentiation are highly conserved among the vertebrates [29]. However, no *DHH *orthologue has been identified in birds, although a paralogue (annotated as DHH) is present in the zebrafish (*Danio*), anole lizard (*Anolis*) and African clawed frog (*Xenopus*) genomes http://www.ensembl.org. Here we describe high conservation of *DHH*, *PTCH1 *and *2 *in the genome of a marsupial, the tammar wallaby (*Macropus eugenii*). Marsupials, have been evolving independently of eutherian mammals for 130- 148 million years [30]. To determine when *DHH *evolved its role in mammalian testicular development, we examined the expression of *DHH *and its receptors *PTCH1 *and *2 *during marsupial gonad development.

## Methods

### Animals

Tammar wallabies of Kangaroo Island, South Australia origin were maintained in our breeding colony in open grassy enclosures. Husbandry, handling and experiments were in accordance with the National Health and Medical Research Council of Australia/Commonwealth Scientific and Industrial Research Organization/Australian Research Council (1990) guidelines and approved by the University of Melbourne Animal Experimentation Ethics Committees.

Pregnancies were initiated by removal of the pouch young (RPY) from animals carrying a blastocyst in embryonic diapause [31,32]. The day of RPY is designated Day 0 of pregnancy and births occur 26-27 days later [32]. Fetuses were removed from the uterus, dissected and tissues snap frozen in liquid nitrogen for mRNA expression analyses or fixed in 4% paraformaldehyde overnight at 4°C for protein localization. The sex of the fetus or pouch young was determined by the presence of scrotal bulges in males or mammary primordia in females [33] or by PCR for SRY [34]. The developing gonadal ridge first becomes apparent around day 22 of gestation in the fetal tammar wallaby [35]. We examined gonads from day 23 of gestation through to adulthood in both males and females.

### Cloning of a tammar wallaby *Dhh, PTCH1 *and *PTCH2*

A full length *DHH *cDNA clone was isolated from a lambda-Zap II cDNA library (constructed from total RNA combined from male and female day 5 postpartum pouch young). The cDNA library was constructed by Clontech Laboratories. Libraries were plated to a density of 120, 000 plaque-forming units per 22 × 22 cm Nunc plate. The library was screened with a full-length mouse Dhh cDNA probe at 60°C in Church's buffer [36].

*PTCH1 *and *2 *sequences were partially isolated from the tammar wallaby genome trace-archives http://www.ensembl.org. PCR and RACE was used to fully characterise the open reading frame (primers listed in Additional file 1) of each receptor. PCR fragments were cloned into p-GEM-T-Easy plasmid (Promega) and sequenced to verify. RACE was performed according to manufacturers instructions (Roche).

### Phylogenetic analyses

Complete cDNA sequences for annotated, full-length hedgehog and patched family members were obtained from the Ensembl database (Ensembl identification numbers listed in Additional file 2) and aligned using MUSCLE http://www.ebi.ac.uk. Trees with fewer than 20 nodes were constructed by aligning sequences using MUSCLE and assembling tree with Mr.Bayes 3.1.2 at 100, 000 generations, sampled every 100 with burnin set to the first 250. Larger trees were aligned with TCoffee http://www.ebi.ac.uk and assembled with Mr.Bayes 3.1.2 at 1, 000, 000 generations, sampled every 1000 with burnin set to the first 250. All trees were viewed and ordered in FigTree 1.3.1.

### RNA extraction

Total RNA was extracted from frozen tissues using the GenElute Total RNA mini kit (Sigma). RNA was DNase treated using the DNAfree reagent (Ambion). DNA free RNA was reverse transcribed using a dT primer and the superscript III kit according to manufacturers instructions (Invitrogen). All RT-PCR derived products were sequence verified as described above.

### Immunohistochemistry

Tissues were fixed in 4% PFA overnight and embedded in paraffin wax according to standard methods. Deparaffinised tissue sections (8 μm) were treated with 3% hydrogen peroxide in methanol for 5 min to quench endogenous peroxidase activity. Antigen retrieval was achieved by placing slides in boiling 0.05 M Tris HCL pH 9.0 for 20 min. Primary antibodies were applied to sections as follows; DHH (anti-mouse DHH, goat polyclonal IgG, R&D Systems, cat.#AF196), 1:400, PTCH1 (anti-human PTCH1, rabbit polyclonal IgG, Abcam, cat.#AB39266) 1:400 and PTCH2 (anti-human PTCH2, rabbit polyclonal IgG, Lifespan BioSciences, cat#LS-B301) 1:400. The DHH antibody was verified by Western Blot to detect a single protein of 43 kDa (the predicted size of the tammar wallaby DHH protein; result not shown Additional file 3). Furthermore, DHH is the primary hedgehog ligand expressed in the marsupial gonad [37]. While the PTCH1 and 2 antibodies were not validated by western blot in the tammar wallaby, the PTCH1 and PTCH2 antibodies were raised against epitopes that share 99 and 100% homology respectively with the analogous tammar target proteins, but that show no cross reactivity. In addition, no other homologous sequences exist within the tammar genome with which the antibody could non-specifically cross react [38]. Primary antibodies were incubated at 4°C overnight. Depending on the primary antibody, the secondary antibody was either an anti-goat IgG raised in rabbit (Millipore) or an anti-rabbit IgG raised in goat (Santa Cruz Biotechnology), both conjugated to biotin and used at a 1:250 dilution. Signal was amplified using the ABC kit (Vector Labs) and visualised using 3-amino-9-ethylcarbazole (AEC) (Vector Labs) and sections were counterstained with haematoxylin. Immunohistochemistry was performed on at least 3 independent samples at each stage. Negative controls were performed as above with the primary antibody omitted.

### Fluorescence *In Situ *Hybridisation Mapping

Chromosomes were prepared from peripheral blood according to standard methods [39]. Chromosome *in situ *suppression (CISS) hybridisation of the genomic lambda clones was performed, with minor modifications [40]. The largest *DHH *cDNA clone was labelled with digoxigenin using nick translation. The labelled probe was incubated with 50 ng of tammar wallaby Cot1 DNA before hybridisation to tammar-fixed metaphase chromosome spreads for 48 h at 37°C. Slides were washed in 0.2 × SSC at 60°C and hybridisation detected with anti-DIG-mouse antibody (Serva), followed by tetramethyl rhodamine iso-thiocyanate (TRITC) conjugated goat-anti-mouse antibody (Serva). Following hybridisation, the slides were counterstained with DAPI (4, 6-diamidino-2-phenylindole) to visualise the chromosomes.

### Expression analyses

RT-PCR was carried out according to standard methods using oligo dT primed cDNA (superscript III, Invitrogen) and 30 PCR cycles. No-template negative controls were included in each round, and in each case showed no amplification (data not shown). A single sample was used for each amplification.

Quantitative PCR was similarly performed on oligo dT primed, reverse transcribed mRNA was prepared as outlined above. Quantitative PCR was carried out using the IQ Sybr green master mix (BioRad) in 20 ul reactions using primers for tammar *DHH, PTCH1 and PTCH2 *(Table 1) and normalised to β-actin as previously described [41]. Each PCR was performed on a single sample in triplicate (except for the adult ovary which was verified on three independent samples) and only those samples with a standard deviation of less than 1 cycle considered for analyses. Relative changes in gene expression were analysed using the methods described by Pfaffl [39,42]. The variance in cycle threshold (ΔCt) was normalised across samples (with beta actin levels set to 100%) and inversely plotted as a percentage of the earliest ΔCt.

## Results

### Isolation of the tammar wallaby *DHH *cDNA

Eight independent cDNA clones of *DHH *were isolated from the tammar wallaby pouch-young cDNA library. The two cDNA clones containing the longest 5' and 3' UTRs and overlapping with each other in the coding region were sequenced. The combined length of the two *DHH *clones was 2.38 kb with a predicted open reading frame of 1, 197 bp. This encoded a protein of 399 amino acids with a predicted molecular weight of 44 kD. There were several common features between the predicted tammar *DHH *protein and that of mouse [43]. A short stretch of N-terminal residues (25 for the tammar and 22 for the mouse) were highly hydrophobic and presumed to function as a signalling peptide. There was a conserved 6 amino acid stretch, CGPGRG, after the signal peptide used to generate the secreted form of DHH. *Drosophila *hedgehog protein and vertebrate Shh, and Dhh proteins are processed into two smaller secreted peptides by an auto-proteolytic process, both *in vitro *and *in vivo *[44]. The catalytic site, GCF, was conserved in the tammar DHH protein, suggesting a similar auto-proteolytic mechanism may also occur to produce a 19.9 kD N-terminal peptide (amino acids 1-200) and a 21.6 kD C-terminal peptide (amino acids 201-403). Furthermore, the tammar and mouse DHH proteins share 96% identity in the N-terminal peptide and 94.5% in the C-terminal peptide, implying that both regions have been highly conserved during evolution (Additional file 4).

*PTCH1 *and *2 *were partially isolated from the tammar wallaby genome http://www.ensembl.org. PCR and RACE was used to fill in missing portions to complete the open reading frame for each gene. *PTCH1 *was seven amino acids longer than its human orthologue and shares 83% sequence homology and 96% amino acid similarity. Conservation was particularly high (85%-100%) in the transmembrane domains and there was complete conservation of the putative glycosylation sites (Additional file 5).

**Figure 1:**
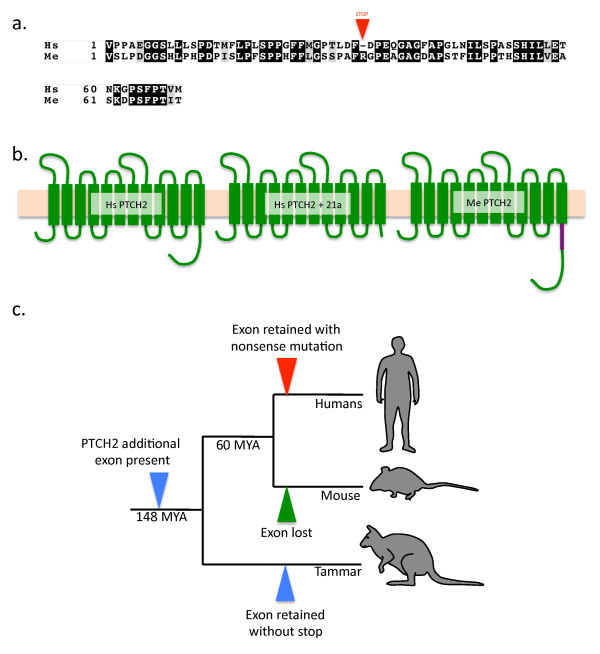
**The structure and evolution of PTCH2 in therian mammals.** a. Amino Acid alignment of the additional PTCH2 tammar wallaby (Me) exon 21a with sequence derived from intron 21 in humans (Hs). The red arrowhead indicates the location of the premature stop codon (-) at amino acid 35 in humans. Black shading indicates identical amino acids, grey shading indicates like amino acids and no shading indicates divergent amino acids. Numbers indicate amino acid position. b. Schematic of the PTCH2 protein derived from the native human isoform (left) the Δ-22 PTCH2 isoform (middle) and tammar PTCH2 with the additional exon 21a (right). c. Phylogenetic tree showing the evolution of exon 21a in mammals. Exon 21a was present in the common therian ancestor (blue arrowhead). The exon was retained without a nonsense codon in the marsupial lineage (blue arrowhead), was mutated in humans to contain a premature stop resulting in the Δ-22 isoform (red arrowhead) and was lost in the rodent lineage (green arrowhead).

*PTCH2 *was more divergent, but still shared 79% sequence identity and 89% amino acid similarity with eutherian orthologues. The tammar PTCH2 predicted protein was 11 amino acids shorter at the N-terminus compared to eutherian PTCH2. In addition we identified a 70 amino acid contiguous insertion immediately downstream of the 12th (last) transmembrane domain located at the C-terminus, which was present in cDNA isoforms isolated from both the day 3 and day 14 post partum (pp) testis (Figure 1). A relaxed Blast search of the non-redundant protein and nucleotide database with this 70 amino acid (210 base pair) sequence failed to return any homology in any described species, except for hits to human and opossum PTCH2 in the same location, downstream of the terminal transmembrane domain. The insertion shows 70% nucleotide identity and 79% amino acid similarity with human. Interestingly, one of the base changes in the human sequence (corresponding to amino acid 35) produced a premature stop codon (Figure 1a). As a result, there were no human protein hits that included this region, only nucleotide homology, and no known ESTs mapping to the nucleotide stretch. The sequence maps to reads in the unannotated genome archives of the opossum with 81% nucleotide identity and 77% amino acid similarity. The sequence shows low homology (< 30% contiguous nucleotide similarity) to mouse and cow gDNA in the same region. Additional exon aside, the C-terminus was the most variable region both within and between eutherians and marsupials (Additional file 6a). As with PTCH1, the transmembrane domains and glycosylation sites were all extremely conserved with 90-100% sequence identity within these regions. Additionally, multiple truncated splice variants of PTCH2 showing developmentally-regulated expression were identified during the subcloning process (Additional file 6b).

### Phylogenetic relationships of DHH, PTCH1 and PTCH2 to eutherian and non-mammalian vertebrates

A phylogenetic analysis of DHH sequences grouped them into four main clusters, one for primates, another elephant, mouse, and hyrax, a third for pig, dog, and dolphin; tammar was an outlier (Additional file 7a). These groupings are largely consistent with the accepted mammalian phylogenetic tree and all eutherian clusters show equal divergence.

**Figure 2:**
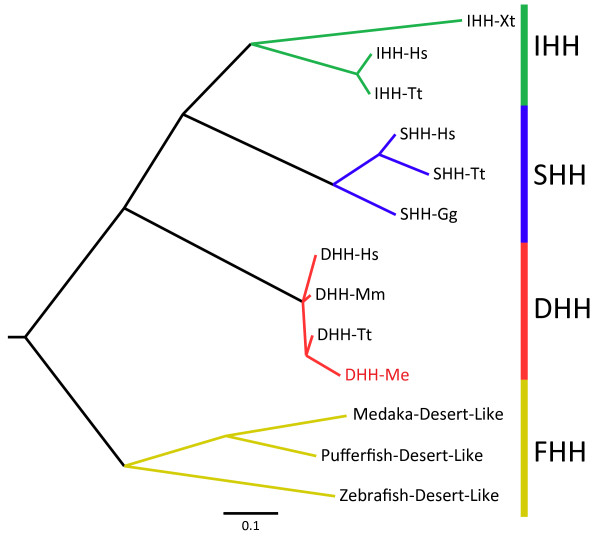
**Phylogenetic tree showing divergence of the hedgehog family members in model organisms in which the genes have been completely sequenced**. Mm = mouse, Me = tammar wallaby, Tt = dolphin, Gg = gorilla, Hs = human, Xt = Xenopus. Complete node labels and distances can be found in additional file 8. Each hedgehog subtype (IHH, SHH, DHH) forms a separate lineage. The fish DHH orthologues form a separate cluster from the mammalian DHH genes and have been renamed the fishy-hedgehog (FHH) cluster.

*DHH *orthologues are absent from the bird genome, but are annotated in other non-mammalian vertebrates including the anole lizard, African clawed frog, zebrafish, medaka, stickleback and puffer fish. To determine the evolutionary relationship of these orthologues to mammalian *DHH *we constructed a phylogenetic tree (as described above) using complete sequences for *SHH*, *IHH *and *DHH *for all vertebrate species, as well as for the zebrafish specific echidna (*EHH*) and tiggywinkle (*TWHH*) hedgehog genes [45]. Our analyses show that the non-mammalian *DHH *orthologues do not cluster with the *DHH *of mammals, but instead form a highly divergent out-group, suggesting that these paralogues had an independent evolutionary origin (Figure 2; Additional file 8) and are hereafter referred to as the *fishy hedgehogs *(*Fhh*).

The PTCH analyses were less inclusive as only a few species had complete open reading frame sequences available. However, the *PTCH1 *phylogenetic tree mimicked standard mammalian groupings, with the tammar clustering with other mammals, and zebrafish as the outlier (Additional file 7b). Phylogenetic analysis of PTCH2 grouped tammar PTCH2 with that of other mammals, but as the most divergent lineage. PTCH2 was more divergent between species than DHH or PTCH1 (Additional file 7c).

All full-length proteins used for phylogenetic analyses were obtained from Ensembl and are listed in Additional file 2.

### Genomic localisation of the tammar wallaby *DHH *cDNA

**Figure 3:**
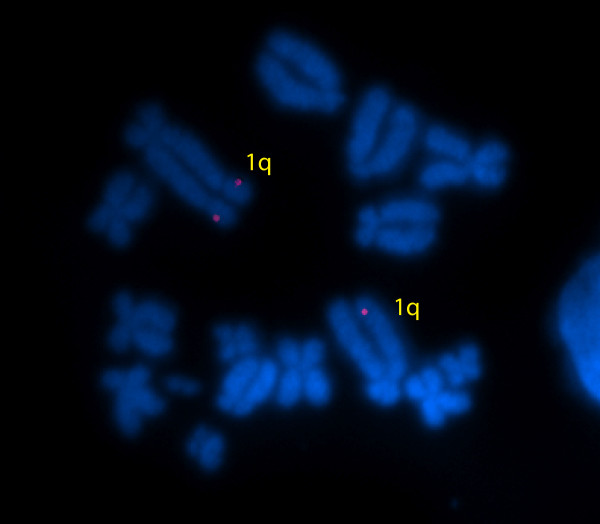
**Fluorescence *in situ *hybridisation of the tammar wallaby DHH cDNA to metaphase chromosome spreads**. The DHH gene maps to chromosome 1q, consistent with its autosomal localisation in eutherian mammals.

A single localization signal was observed for tammar wallaby *DHH *on chromosome 1q in approximately 50% of spreads examined, with no other consistent localization signals seen (Figure 3).

### Expression of *DHH *in the tammar wallaby

**Figure 4:**
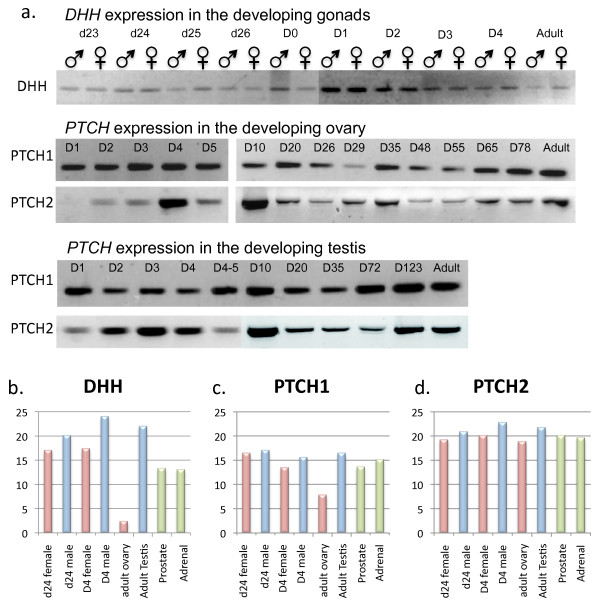
**Expression of DHH, PTCH1 and PTCH2 in the tammar wallaby.** a. RT-PCR for *DHH*, *PTCH1 *and *PTCH2 *in the tammar wallaby testis and ovary throughout development. *DHH *primers produce a band of 365 bp, *PTCH1 *primers produce a 130 bp product and *PTCH2 *primers produce a 330 bp product. d = day of fetal development from a 26.5 average gestation period. D0 = the day of birth. D = day of development post partum (pp). *DHH *expression was present at all time points during ovarian development. b-d. Quantitative PCR for DHH, PTCH1 and PTCH2. b. DHH was expressed at high levels in the developing gonads of both males and females (although it was lower in females overall). The lowest level of expression was observed in the adult ovary and the highest in the D4 male. Both PTCH1 (c) and PTCH2 (d) levels remained relatively consistent throughout gonad development. PTCH2 mRNA levels were always higher than PTCH1 in the gonads, and levels were consistently lowest for both genes in the adult ovary.

*DHH, PTCH1 *and *PTCH2 *expression was examined in the gonads throughout fetal and pouch young development using an RT-PCR assay. In contrast to the mouse, *DHH *was expressed in the gonads of both male and female fetuses and pouch young. *DHH *was expressed from the first appearance of the genital ridge until after testicular and ovarian differentiation had occurred and persisted in the adult (Figure 4a). In addition, both *PTCH1 *and *PTCH2 *were expressed throughout gonadal development, through to adulthood. Although not quantitative, *PTCH2 *expression appeared to be expressed at a lower level than *PTCH1*. In the testis, both *PTCH1 *and *PTCH2 *were present from 1 day after birth, around the time of initiation of cord formation in the tammar (Figure 4a).

A limited quantitative real-time PCR profile confirmed the presence of *DHH *at all stages of gonad development in both testes and ovaries (Figure 4b-d). *DHH *mRNA levels were similar in the developing male and female gonads at all stages examined, but in adult gonads ovarian expression was low (Figure 4b). *PTCH1 *expression was consistent throughout gonadal development in males and females, with lowest levels seen in the adult ovary (Figure 4c). In contrast, *PTCH2 *levels remained constant in the gonad of both males and females at all stages examined including in the adult (Figure 4d). All three genes were also expressed in the prostate and adrenal albeit at lower levels than in the developing gonads.

### DHH, PTCH1 and PTCH2 protein distribution during gonadogenesis

**Figure 5:**
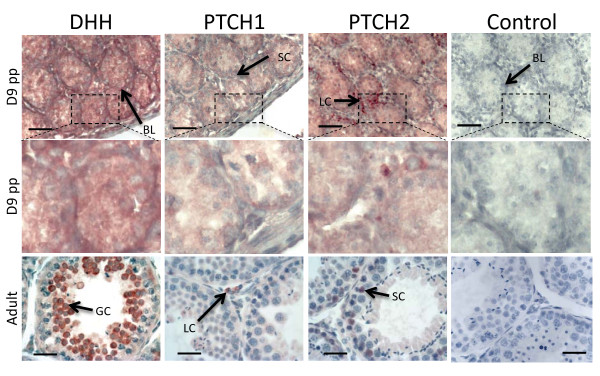
**Immunohistochemistry of DHH, PTCH1 and PTCH2 in the tammar wallaby testis at key developmental time points**. Red/brown staining indicates protein distribution while the heamatoxalin counterstain appears blue. It is important to note that DHH is a highly secreted molecule and staining does not imply cell of origin. DHH staining was most intense at the basal lamina (BL). In the adult staining is concentrated in the develop spermatocytes (GC). At day D9pp PTCH1 was present within the Sertoli cells (SC) while PTCH2 was predominant in the Leydig cells (LC). This expression profile is reversed in the adult testis with PTCH1 found predominantly in the Leydig cells, while PTCH2 was predominate in the Sertoli cells. Scale bars indicate 40 μm, controls show immunohistochemistry with the primary antibody omitted.

DHH was widely distributed throughout the bipotential gonad, PTCH1 and 2 staining was present but very weak (Additional file 9, d24 fetus). By day 1 pp the cords were beginning to form in the testis, and DHH stained pre-Sertoli cells that were coalescing into cord- like structures (Additional file 9, D1pp). PTCH1 protein stained weakly throughout the gonad while PTCH2 appeared more prominent and was localized outside the forming cords. At day 9 pp cords were fully formed in the developing testis. DHH protein was present throughout the gonad and was strongly detected in some Sertoli cells and in the peritubular myoid cells and at the basement membrane. PTCH1 was diffuse throughout the gonad but was mainly localised in the Sertoli cells and absent from the interstitium. Conversely, PTCH2 staining was more intense and concentrated in Leydig cells in the interstitium (Figure 5). In the adult testis, DHH was present at low levels in all cell types, but strong staining was seen in round spermatids from the post pachytene primary spermatocyte stage, through to the mature sperm. PTCH1 was present at high levels in the Leydig cells and showed a punctate distribution reminiscent of membrane bound protein recycling [46]. PTCH2 distribution also became highly restricted and localised strongly in the Sertoli cells (Figure 5).

**Figure 6:**
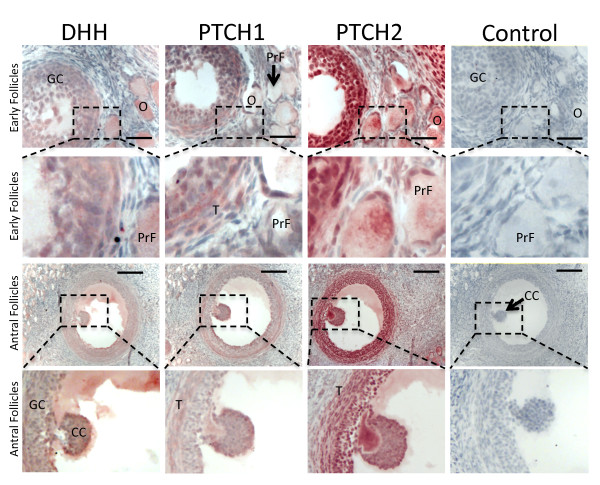
**Immunohistochemistry of DHH, PTCH1 and PTCH2 in the tammar wallaby ovary**. DHH was found in the granulosa cells (GC) of follicles at all stages of development, and in the oocyte cytoplasm shown in primordial follicles (PrF). Staining was also observed for all three proteins in the cumulus cells (CC). PTCH1 and PTCH2 showed a similar distribution in the granulosa cells and were also present at reduced levels in the theca (T). PTCH2 staining was evident in the oocyte but PTCH1 was not. Scale bars indicate 160 μm, controls show immunohistochemistry with the primary antibody omitted.

The ovary becomes clearly differentiated around day 8 pp in the tammar [35]. At day 9 pp DHH staining was diffuse throughout the gonad along with PTCH1 and PTCH2 (Additional file 10). However, all three proteins were noticeably absent from the germ cells. By day 72 pp DHH was present in low levels across the ovary, but strongly localised within the germ cells that have coalesced into nests. PTCH1 was also expressed in the germ cells at this stage, while PTCH2 staining was weak and primarily in the interstitial tissue (Additional file 9). In the adult ovary, DHH was present but weak throughout the gonad. PTCH1 staining was only weakly detected in the granulosa cells of antral follicles, but increased in the cumulus cells of mature follicles. Staining was also seen in the steroidogenic theca cells. PTCH2 showed a similar distribution to PTCH1 but was abundant in the granulosa, cumulus and theca cells (Figure 6). DHH, PTCH1, and PTCH2 were also detected in the corpus luteum (Additional file 9).

## Discussion

We have shown that *DHH *is a highly conserved mammal specific hedgehog paralogue with conserved expression during mammalian gonadogenesis. DHH and its receptors PTCH1 and 2 are highly conserved at the protein level and are expressed in an analogous pattern to that seen in the mouse gonad. However, *DHH *was expressed in the developing marsupial ovary in contrast to the mouse, in which it is testis-specific during development.

Phylogenetic analysis of the hedgehog gene family across vertebrates shows that non-mammalian DHH genes in fish form a distinct subgroup, distantly related to mammalian DHH genes, indicating they have had an independent evolutionary origin. We have re-named this sub-group fishy hedgehog (FHH) to emphasise their distinction from the DHH genes. This suggests that the evolution of mammalian *DHH *is a recent event (Figure 2) making it quite unique among the gonadal differentiation genes, all of which have orthologues in the non-mammalian vertebrates with the notable exception of the sex determination switch gene *SRY*, which is also mammal specific [47]. Despite its recent origin, *DHH *was extremely highly conserved between marsupials and eutherians, suggesting it quickly adopted an essential function in mammalian reproduction.

The hedgehog receptors *PTCH1 *and 2 were highly conserved between marsupials, eutherians and non-mammalian vertebrates. Marsupial *PTCH2 *was the most divergent (especially in the C-terminal region, consistent with findings in other vertebrate species [48]) but still shared 89% amino acid similarity with eutherian orthologues. The tammar *PTCH2 *C-terminus contained a 70 amino acid additional exon not found in eutherian PTCH2 proteins. Interestingly, significant homology to the additional tammar exon was identified in the human *PTCH2 *genomic sequence, in intron 21, and shared 70% identity at the nucleotide level and 79% amino acid similarity with the tammar additional exon (hereafter referred to as exon 21a). The level of conservation of this exon between marsupials and humans was much higher than that of non-functional intronic DNA, suggesting functional conservation of the sequence. Translation of the human sequence revealed a premature stop codon at amino acid 35, so its inclusion in the transcript would lead to a *PTCH2 *receptor with a severely truncated intracellular signalling domain (Figure 1b). Such an isoform, lacking exon 22, identical to the one predicted from the inclusion of the human putative exon 21a, has been previously identified (the Δ-22 isoform)[18]. The human Δ-22 PTCH2 isoform is the only one capable of acting as a strong inhibitor of SHH induction, similar in function to PTCH1 [18]. It appears that the ability to produce such an isoform was derived from a stochastic nonsense mutation in the original exon 21a leading to a truncated protein. The tammar does not have a premature stop (exon 21a is an intact ORF) and so this tammar PTCH2 isoform does not share redundancy with PTCH1 function. The degree of conservation of this region in humans suggests that it has only recently become non-functional in primate evolution. It is intriguing then, that this sequence could not be identified in any other mammalian *PTCH2 *loci, but only in the tammar, opossum, and human. These findings suggest that the exon was present in the ancestral *PTCH2 *gene and has been independently lost in different eutherian lineages (Figure 1c). We also identified several *PTCH2 *isoforms that appear to be dynamically regulated at specific developmental time points. This is also consistent with findings in humans that identified *PTCH2 *isoforms lacking exons 9 and 10 (PTCH2-Δ9-10)[18]. Taken together, these data suggest that *PTCH2 *has divergent species-specific roles in development, while *PTCH1 *is likely to maintain a highly conserved function in hedgehog signal transduction. Furthermore, it suggests that the human PTCH2 Δ-22 isoform may have evolved to compensate for a loss of PTCH1 in tissues in which they are co-expressed.

*DHH, PTCH1 and PTCH2 *mRNA and protein were present throughout gonadal development in both males and females, from early development through to adult stages. The presence of ligand and both receptor proteins throughout gonadal development is consistent with findings in mouse testis, but not ovary [49] and suggests a conserved role for hedgehog signalling in mammalian gonad formation. These findings are also consistent with the observed disruption to normal gonadal patterning and significant reduction in the expression of the downstream target gene *GLI1*, in the tammar when hedgehog signalling is ablated *in vitro *[37].

In the testis, DHH could be seen within the pre-Sertoli cells of the aggregating cords. Once the testes differentiated, DHH staining was concentrated in the Sertoli cells, especially at the basal lamina of the cord. This protein distribution is similar to that reported in mouse [49,24,49] and suggests it is critical for testicular patterning. However, there were some differences in *PTCH *distribution from predicted mouse patterns. PTCH1 staining was similar to that of DHH, and was distributed mainly within the seminiferous cords (containing germ cells and Sertoli cells). This was in contrast to the interstitial expression seen for *Ptch1 *in the developing mouse testis [24] but similar to the expression of *Ptch2 *[17]. Conversely, PTCH2 staining in the tammar was more reminiscent of Ptch1 distribution in the mouse testis [17] and was located throughout the gonad but concentrated in the interstitium and Leydig cells. This suggests there may have been a reversal in the roles of these receptors in marsupial testicular development relative to the mouse. Since detailed localisation of the PTCH receptors during gonad development in other mammals and vertebrates is not available we cannot determine which profile is more typical during development.

There was discrete staining of DHH, PTCH1 and PTCH2 proteins in the adult testis. DHH was concentrated in the differentiating germ cells, but restricted to the post pachytene primary spermatocyte stage through to the mature sperm. There was faint PTCH1 and PTCH2 staining throughout the testis but the proteins were concentrated in the Leydig cells and Sertoli cells respectively. This is consistent with *in situ *results in the adult mouse testis [17], suggesting a conserved role for these genes in maintaining testicular function and spermatogenesis in all therian mammals.

Unlike in the mouse, in which *Dhh *is testis-specific in early development [1], *DHH *was expressed in the developing tammar ovary throughout development. Activation of hedgehog signalling in the developing mouse ovary leads to Leydig cell development [25]. However, early ovarian development was not affected by the presence of *DHH *in the tammar, despite the presence of similar mRNA and protein levels of both ligand and receptors as in the developing testis. These findings show that SRY is not needed for *DHH *activation in the developing gonad. In the juvenile ovary, DHH was abundant in the oocytes consistent with the suggested role for DHH in maintaining the germ line [1]. In the adult ovary, DHH was broadly co-localized with PTCH1 and 2, in follicles and the corpus luteum suggesting it may be important for normal folliculogenesis and steroidogenesis, consistent with recent findings in the mouse [26]. As in the testis, PTCH2 appeared to be the predominant receptor throughout ovarian development and in the adult.

## Conclusions

These data support a conserved role for hedgehog signalling in gonadal development but show that in marsupials this pathway may be significant for early patterning of the ovary as well as for the testis. These results, in conjunction with our phylogenetic analysis of hedgehog family members in all vertebrates, suggests that *DHH *is unique to mammals and is a conserved member of the gonadal development pathway.

## Authors' contributions

All authors participated in the design of the study. Tissues were collected by WJA, AJP and MBR. Experiments were performed by WAO'H, WJA and AJP. All authors analyzed the results. AJP, WJA, WAO'H and MBR drafted the manuscript. All authors read, modified and approved the final manuscript.

## Supplementary Material

Additional file 1 **Primes used to PCR clone and check splice variants of the genes described**. Click here for file

Additional file 2 **Table of full-length hedgehog and PTCH sequences used for phylogenetic analyses**. Click here for file

Additional file 3 **a. Schematic diagram of the alternative splice variants detected for tammar wallaby *PTCH2 *relative to the human *PTCH2 *structure. Primers spanned exons 16-22 (red bar) and 7 splice variants (including the full length transcript) were isolated**. Tammar *PTCH2 *has two additional introns in exon 17 and 22 (blue arrow heads) and one additional exon (21-Me; red arrow). b. Table showing the relative homologies of the epitope to which the DHH antibody was raised (recombinant mouse (Rm) Dhh amino acids 199-396) to tammar wallaby DHH, SHH and IHH. Homology is significantly lower with SHH and IHH. c. Western Blot of DHH antibody a band at 43 kDa, which is the predicted size of the tammar wallaby DHH protein in its uncleaved form. Antibody cross-reactivity with SHH or IHH would create bands at 48 and 45 kDa respectively. Click here for file

Additional file 4 **Alignment of tammar Dhh protein sequence with four eutherian mammals**. Dark shading indicates agreement in at least 60% of the sequences, light shading indicates amino acid similarity to consensus. Double dashed area represents conserved sequence necessary for secreted DHH. Asterisked region represents conserved catalytic site. Click here for file

Additional file 5 **Alignment of tammar Ptch1 protein sequence with four eutherian mammals**. Dark shading indicates agreement in at least 60% of the sequences, light shading indicates amino acid similarity to consensus. Double dashed areas represent putative trans-membrane binding domains, with species (Ptch1 unless indicated) showing highest sequence identity indicated in parentheses. Any conserved domains are mentioned above the relative sequence. Glycosylation sites are denoted with a cross. Click here for file

Additional file 6 **a. Alignment of tammar Ptch2 protein sequence with four eutherian mammals**. Dark shading indicates agreement in at least 60% of the sequences, light shading indicates amino acid similarity to consensus. 70 amino acid stretch maintained in Tammar is italicized. Double dashed areas represent putative trans-membrane binding domains, with species showing highest sequence identity indicated in parentheses. Any conserved domains are mentioned above the relative sequence. b. Alternative splice variants of PTCH2. Primers were designed to span the region corresponding to exons 18-22 of the human PTCH2 gene. RT-PCR was carried out in day 3, 7 and 14 post partum testes. Day 3 PCR produced four bands of ~1.2 Kb, 1.05 Kb, 950 bp and 860 bp. We sequence verified that the 1.2 Kb fragment was the full-length transcript and that the 950 bp transcript was a Δ-21a PTCH2 isoform. The identity of the missing exons in the 1.05 Kb and 860 bp fragments is shown in Additional File 9. These slice variants were developmentally regulated, with the smaller two isoforms not seen in the day 7 or 14 testis and the larger two isoforms appear to change in their relative abundance between stages. Click here for file

Additional file 7 **a-c. Phylogenetic trees showing divergence of DHH(A), PTCH1(B), and PTCH2(C) in model organisms in which the genes have been completely sequenced**. Mm = mouse, La = elephant, Me = tammar wallaby, Cf = dog, Tt = dolphin, Ss = pig, Gg = gorilla, Hs = human, Pp = Chimpanzee, Bt = cow, Dr = zebrafish, Tn = Tetraodon, Ol = Oryzias. Zebrafish is included in b and c as a known outlier. Click here for file

Additional file 8 **Phylogenetic tree showing clustering of all complete sequenced HH proteins (Indian (IHH), Sonic (SHH), desert (DHH), Echidna (EHH), TwiggyWinkle (TWHH))**. EHH and TWHH each contain only 1 member, and have both been shown to cluster within IHH and SHH groups respectively. The fish DHH orthologues (FHH) form a separate cluster from the mammalian DHH genes. EHH, TWHH and reported fish DHH orthologues (FHH) are highlighted in red. Node labels are in the format: PROTEIN_Genus_species. Click here for file

Additional file 9 **Immunohistochemistry of DHH, PTCH1 and PTCH2 in the tammar wallaby testis at key developmental time points**. Red/brown staining indicates protein distribution while the heamatoxalin counterstain appears blue. It is important to note that DHH is a highly secreted molecule and staining does not imply cell of origin. DHH was initially present at high levels throughout the indifferent gonad (d24 fetus), by D1pp Dhh is confined to the aggregating seminiferous cords (AC). Scale bars = 36 μm. Click here for file

Additional file 10 **Immunohistochemistry of DHH, PTCH1 and PTCH2 in the tammar wallaby ovary at key developmental time points**. At day 9pp when the ovary is forming a cortex and medulla, there was widespread staining for DHH, PTCH1 and PTCH2 throughout the ovary. By D72pp DHH and PTCH1 were concentrated in the germ cell nests (CGN) and PTCH2 was largely in the interstitium. In the adult ovary, DHH was found in the granulosa cells (GC) of follicles at all stages of development, and in the oocyte cytoplasm. Staining was also observed in the corpus luteum (CL). Scale bars = 40 μm at D9, D72 and 160 μm in the corpus luteum. Click here for file
