# Supplementary material for: Desert hedgehog is a mammal-specific gene expressed during testicular and ovarian development in a marsupial
Source: BMC Dev Biol. 2011 Dec 1;11:72. doi: 10.1186/1471-213X-11-72 (PMC3293750; doi:10.1186/1471-213X-11-72)
Supplement: Additional file 1 — Primes used to PCR clone and check splice variants of the genes described. [file 1471-213X-11-72-S1.PDF]

Primer Name	Application	Sequence
Ptch1/1F	PCR cloning	5-ACTATGCTTCGGTGGGACTG-3
Ptch1/1R	PCR cloning	5-AACTGTGGATTGCATGGTGA-3
Ptch1/2F	PCR cloning	5-TCTGGTGGATGCAGATGGTA-3
Ptch1/2R	PCR cloning	5-GAGCCATTGTTTGTGTGTCG-3
Ptch2/1F	PCR cloning	5-AGAGAAGCTTGGGGAAGAGG-3
Ptch2/1R	PCR cloning	5-GACCTGCCTGCTATGGTGAT-3
Ptch2/2F	PCR cloning	5-CCAAGGAACCCCTGGACTAT-3
Ptch2/2R	PCR cloning	5-GAGGCCGTCAGAGTGGTAAA-3
Ptch2/3F	PCR cloning	5-CGGGAGCTTTACCACTCTGA-3
Ptch2/3R	PCR cloning	5-TTGCATAGAGGCAGGTCCAT-3
MeExon1F	PCR - start codon verification	5-TTTGCGTCTCCCCCGTAGGGC-3
MeExon2R	PCR - start codon verification	5-CCTCTTCCCCAAGCTTCTCT-3
P2Start_Nested	RACE-PCR - UTR sequencing	5-CCCAAAAGCAGTCCAGCGGCGTGAGG-3
P2Start_Outer	RACE-PCR - UTR sequencing	5-GATGCAGACAGGGCCGCCCAACATAG-3
P2Stop_Nested	RACE-PCR - UTR sequencing	5-CCTTCATCCTGCCCCCAACACACTCC-3
P2Stop_Outer	RACE-PCR - UTR sequencing	5-AGGTCCTGAGGCAGGAGCAGGGGATG-3
