# Supplementary material for: Desert hedgehog is a mammal-specific gene expressed during testicular and ovarian development in a marsupial
Source: BMC Dev Biol. 2011 Dec 1;11:72. doi: 10.1186/1471-213X-11-72 (PMC3293750; doi:10.1186/1471-213X-11-72)
Supplement: Additional file 2 — Table of full-length hedgehog and PTCH sequences used for phylogenetic analyses. [file 1471-213X-11-72-S2.PDF]

Species	Gene	Ensembl.org ID
Danio rerio	EHH	ENSDARP00000076199
Danio rerio	TWHH	ENSDARP00000056746
Echinops telfairi	DHH	ENSETET00000001417
Procavia capensis	DHH	ENSPCAP00000003714
Loxodonta africana	DHH	ENSLAFP00000008387
Mus musculus	DHH	ENSMUSP00000023737
Rattus norvegicus	DHH	ENSRNOP00000020563
Gorilla gorilla	DHH	ENSGGOP00000007809
Homo sapiens	DHH	ENSP00000266991
Nomascus leucogenys	DHH	ENSNLEP00000021679
Pongo abelii	DHH	ENSPPYP00000005114
Microcebus murinus	DHH	ENSMICP00000015983
Tursiops truncatus	DHH	ENSTTRP00000004503
Sorex araneus	DHH	ENSSARP00000005929
Pteropus vampyrus	DHH	ENSPVAP00000004390
Myotis lucifugus	DHH	ENSMLUP00000007616
Canis familiaris	DHH	ENSCAFP00000012759
Tupaia belangeri	DHH	ENSTBEP00000001345
Danio rerio	DHH	ENSDARP00000053870
Oryzias latipes	DHH	ENSORLP00000009519
Tetraodon nigroviridis	DHH	ENSTNIP00000003035
Callithrix jacchus	IHH	ENSCJAP00000021087
Homo sapiens	IHH	ENSP00000295731
Macaca mulatta	IHH	ENSMMUP00000001060
Pongo abelii	IHH	ENSPPYP00000014741
Pan troglodytes	IHH	ENSPTRP00000022106
Otolemur garnetti	IHH	ENSOGAP00000012635
Procavia capensis	IHH	ENSPCAP00000010585
Cavia porcellus	IHH	ENSCPOP00000016106
Dipodomys ordii	IHH	ENSDORP00000008328
Mus musculus	IHH	ENSMUSP00000006713
Bos taurus	IHH	ENSBTAP00000053248
Tursiops truncatus	IHH	ENSTTRP00000009502
Macropus eugenii	IHH	ENSMEUP00000003246
Anolis carolinensis	IHH	ENSACAP00000005052
Xenopus tropicalis	IHH	ENSXETP00000030370
Danio rerio	IHH	ENSDARP00000076101
Gasterosteus aculeatus	IHH	ENSGACP00000020527
Tetraodon nigroviridis	IHH	ENSTNIP00000019547
Takifugu rubripes	IHH	ENSTRUP00000030966
Danio rerio	SHH	ENSDARP00000089976
Gasterosteus aculeatus	SHH	ENSGACP00000005121
Gorilla gorilla	SHH	ENSGGOP00000021296
Homo sapiens	SHH	ENSP00000297261
Nomascus leucogenys	SHH	ENSNLEP00000018692
Pongo abelii	SHH	ENSPPYP00000020423
Macaca mulatta	SHH	ENSMMUP00000009646
Mus musculus	SHH	ENSMUSP00000002708
Rattus norvegicus	SHH	ENSRNOP00000008497
Canis familiaris	SHH	ENSCAFP00000007602
Sus scrufa	SHH	ENSSSCP00000017394
Tursiops truncatus	SHH	ENSTTRP00000013015

Species	Gene	Ensembl.org ID
Monodelphis domestica	SHH	ENSMODP00000006183
Gallus gallus	SHH	ENSGALP00000010292
Taeniopygia guttata	SHH	ENSTGUP00000000707
Macropus eugenii	PTCH1	GenBank JF925124
Homo sapiens	PTCH1	ENSP00000364423
Bos taurus	PTCH1	ENSBTAP00000013074
Mus musculus	PTCH1	ENSMUSP00000021921
Pongo abelii	PTCH1	ENSPPYP00000021750
Macropus eugenii	PTCH2	GenBank JF925125
Canis familiaris	PTCH2	ENSCAFP00000007005
Bos taurus	PTCH2	ENSBTAP00000033224
Homo sapiens	PTCH2	ENSP00000361266
Mus musculus	PTCH2	ENSMUSP00000030443
