# Supplementary figures and images for: Desert hedgehog is a mammal-specific gene expressed during testicular and ovarian development in a marsupial
Source: BMC Dev Biol. 2011 Dec 1;11:72. doi: 10.1186/1471-213X-11-72 (PMC3293750; doi:10.1186/1471-213X-11-72)

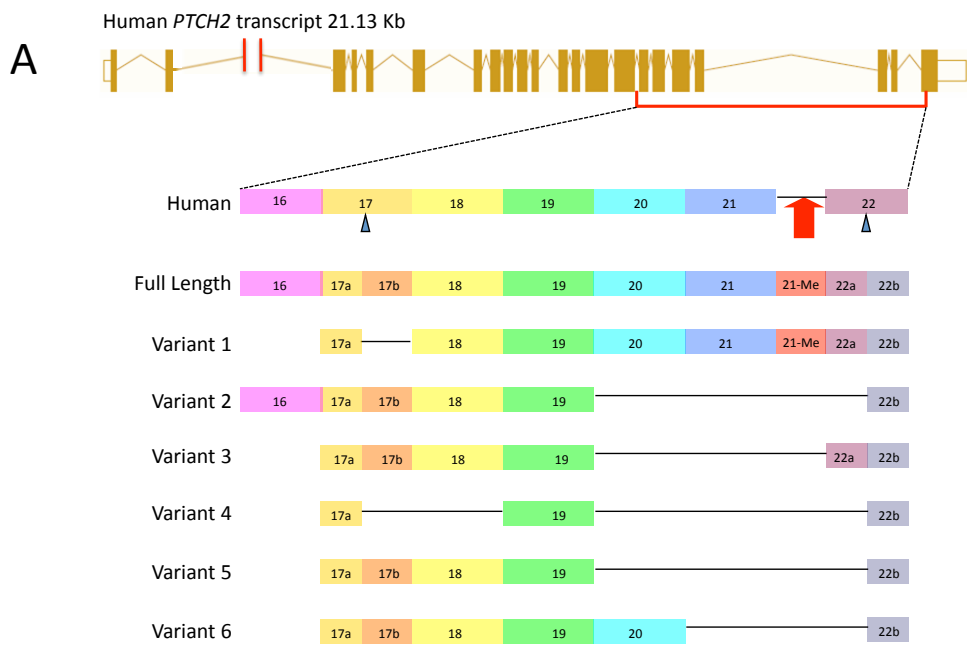

**B**

|              | Me DHH | Me SHH | Me IHH |
|--------------|--------|--------|--------|
| RmDhh        | SI 93% | SI 39% | SI 44% |
| (aa 199-396) | SS 95% | SS 49% | SS 57% |

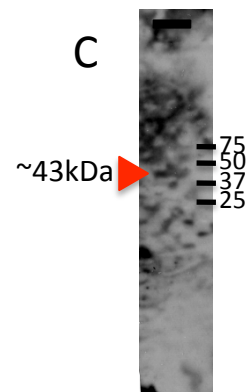

Supplement: Additional file 3 — a. Schematic diagram of the alternative splice variants detected for tammar wallaby PTCH2 relative to the human PTCH2 structure. Primers spanned exons 16-22 (red bar) and 7 splice variants (including the full length transcript) were isolated. Tammar PTCH2 has two additional introns in exon 17 and 22 (blue arrow heads) and one additional exon (21-Me; red arrow). b. Table showing the relative homologies of the epitope to which the DHH antibody was raised (recombinant mouse (Rm) Dhh amino acids 199-396) to tammar wallaby DHH, SHH and IHH. Homology is significantly lower with SHH and IHH. c. Western Blot of DHH antibody a band at 43 kDa, which is the predicted size of the tammar wallaby DHH protein in its uncleaved form. Antibody cross-reactivity with SHH or IHH would create bands at 48 and 45 kDa respectively. [file 1471-213X-11-72-S3.PDF]

a.

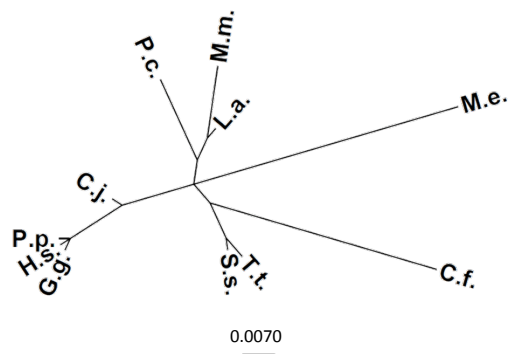

b.

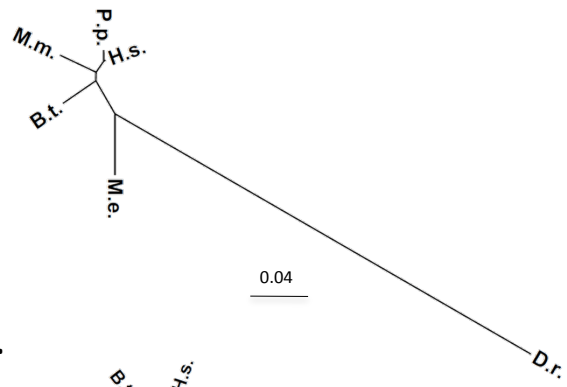

c.

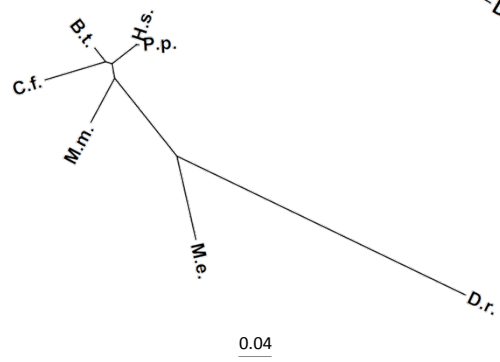

Supplement: Additional file 7 — a-c. Phylogenetic trees showing divergence of DHH(A), PTCH1(B), and PTCH2(C) in model organisms in which the genes have been completely sequenced. Mm = mouse, La = elephant, Me = tammar wallaby, Cf = dog, Tt = dolphin, Ss = pig, Gg = gorilla, Hs = human, Pp = Chimpanzee, Bt = cow, Dr = zebrafish, Tn = Tetraodon, Ol = Oryzias. Zebrafish is included in b and c as a known outlier. [file 1471-213X-11-72-S7.PDF]

# HH Family

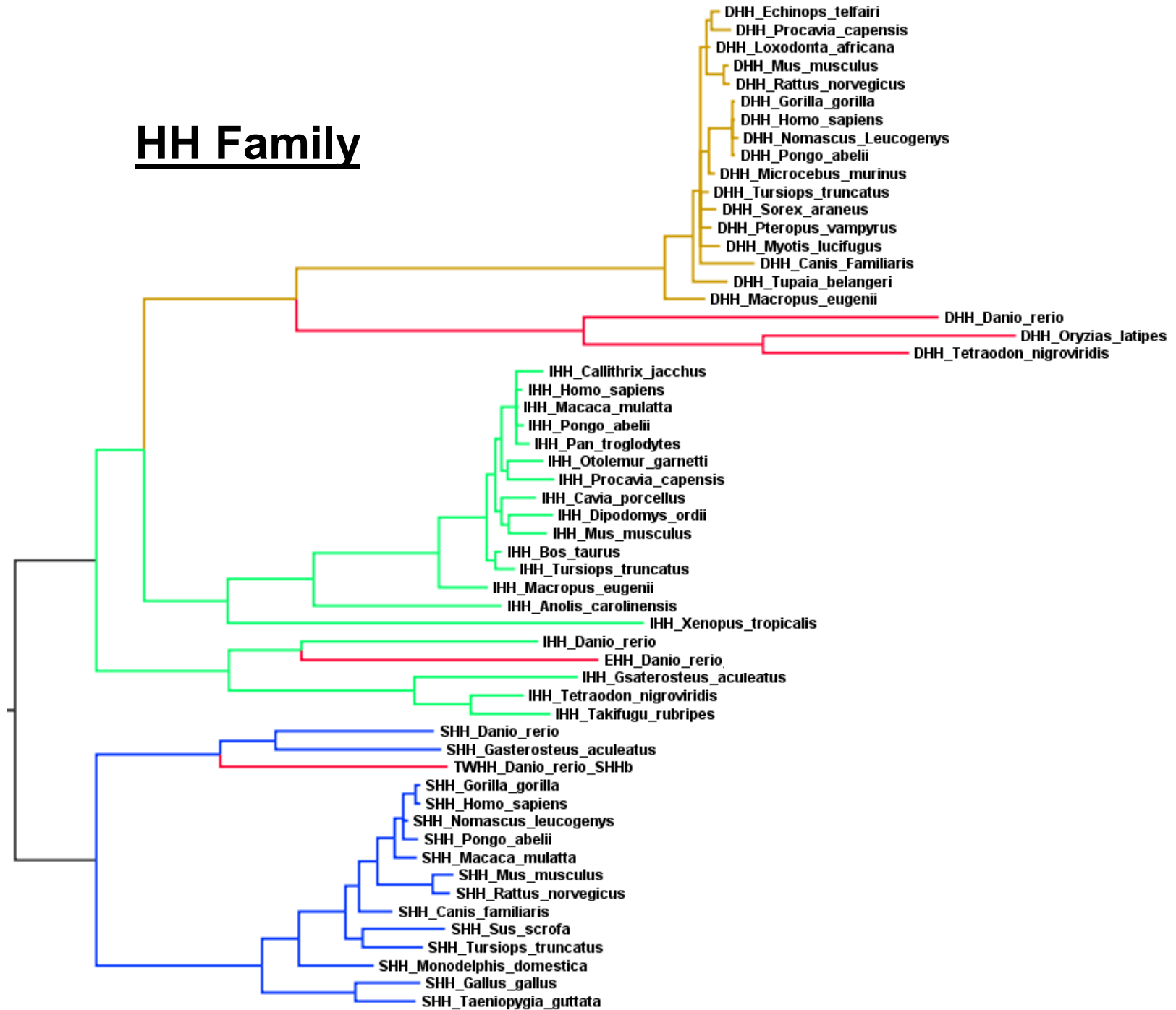

Supplement: Additional file 8 — Phylogenetic tree showing clustering of all complete sequenced HH proteins (Indian (IHH), Sonic (SHH), desert (DHH), Echidna (EHH), TwiggyWinkle (TWHH)). EHH and TWHH each contain only 1 member, and have both been shown to cluster within IHH and SHH groups respectively. The fish DHH orthologues (FHH) form a separate cluster from the mammalian DHH genes. EHH, TWHH and reported fish DHH orthologues (FHH) are highlighted in red. Node labels are in the format: PROTEIN_Genus_species. [file 1471-213X-11-72-S8.PDF]

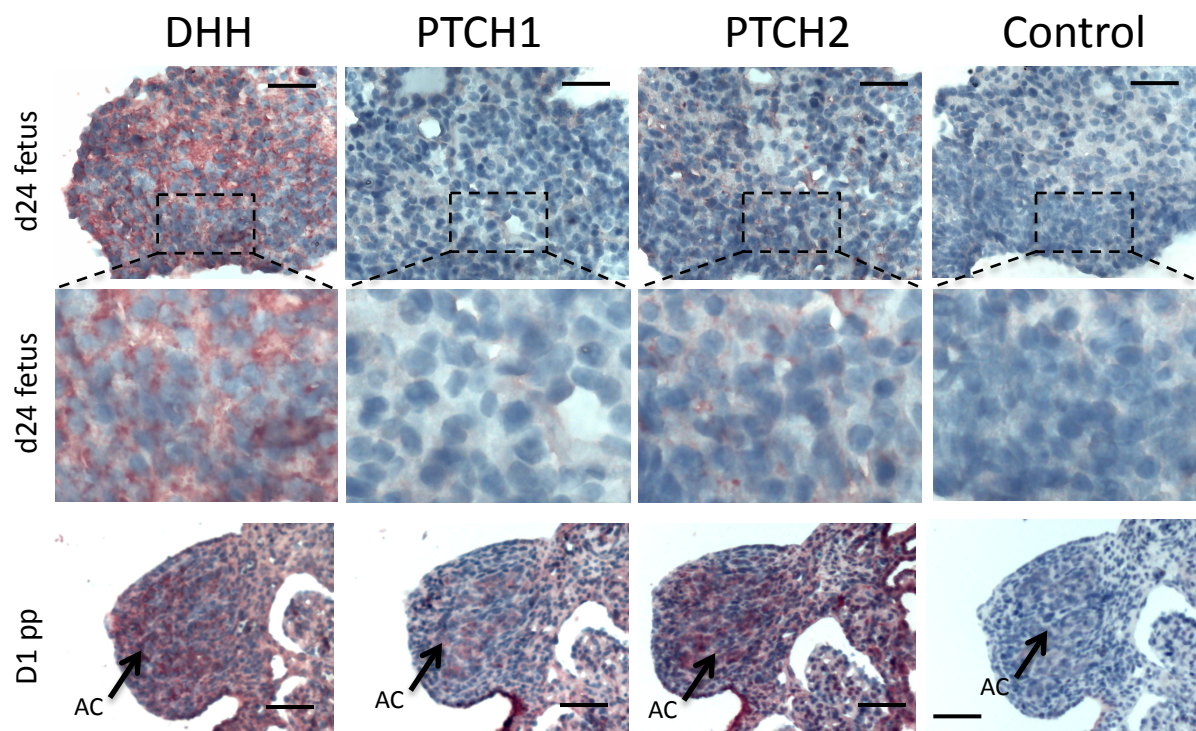

Supplement: Additional file 9 — Immunohistochemistry of DHH, PTCH1 and PTCH2 in the tammar wallaby testis at key developmental time points. Red/brown staining indicates protein distribution while the heamatoxalin counterstain appears blue. It is important to note that DHH is a highly secreted molecule and staining does not imply cell of origin. DHH was initially present at high levels throughout the indifferent gonad (d24 fetus), by D1pp Dhh is confined to the aggregating seminiferous cords (AC). Scale bars = 36 μm. [file 1471-213X-11-72-S9.PDF]

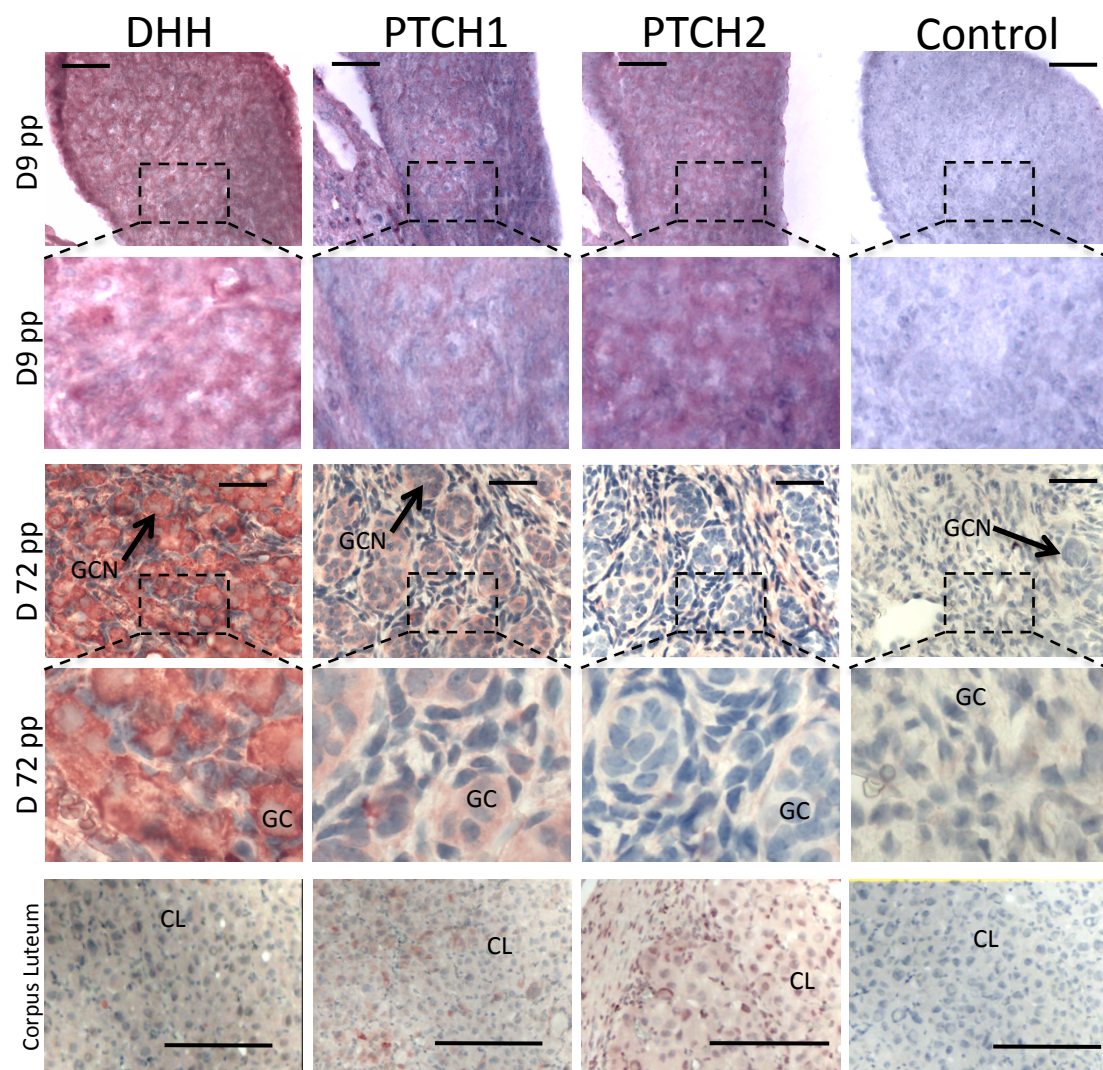

Supplement: Additional file 10 — Immunohistochemistry of DHH, PTCH1 and PTCH2 in the tammar wallaby ovary at key developmental time points. At day 9pp when the ovary is forming a cortex and medulla, there was widespread staining for DHH, PTCH1 and PTCH2 throughout the ovary. By D72pp DHH and PTCH1 were concentrated in the germ cell nests (CGN) and PTCH2 was largely in the interstitium. In the adult ovary, DHH was found in the granulosa cells (GC) of follicles at all stages of development, and in the oocyte cytoplasm. Staining was also observed in the corpus luteum (CL). Scale bars = 40 μm at D9, D72 and 160 μm in the corpus luteum. [file 1471-213X-11-72-S10.PDF]
