# Supplementary material for: Desert hedgehog is a mammal-specific gene expressed during testicular and ovarian development in a marsupial
Source: BMC Dev Biol. 2011 Dec 1;11:72. doi: 10.1186/1471-213X-11-72 (PMC3293750; doi:10.1186/1471-213X-11-72)
Supplement: Additional file 4 — Alignment of tammar Dhh protein sequence with four eutherian mammals. Dark shading indicates agreement in at least 60% of the sequences, light shading indicates amino acid similarity to consensus. Double dashed area represents conserved sequence necessary for secreted DHH. Asterisked region represents conserved catalytic site. [file 1471-213X-11-72-S4.PDF]

```
                                            =====
H.sapiens        1 MALLTNLLPLCCLALLA---LPAQSCGPGRGPVGRRRYARKQLVPLLYKQFVPGVPERTL
P.pygmaeus       1 MALLTNLLPLCCLALLA---LPAQSCGPGRGPVGRRRYARKQLVPLLYKQFVPGVPERTL
C.familiaris     1 MALPARLVPLCCLALLA---LPAQSCGPGRGPVGRRRYVRKQLVPLLYKQFVPSVPERTL
M.musculus       1 MALPASLLPLCCLALLA---LSAQSCGPGRGPVGRRRYVRKQLVPLLYKQFVPSMPERTL
M.eugenii        1 MALRASLVPVCCLALLALLTLPTRSCGPGRGPVGRRRYVRKQLIPLLYKQFVPSVPERTL

H.sapiens       58 GASGPAEGRVARGSERFRDLVPNYNPDIIFKDEENSGADRLMTERCKERVNALAIAVMNM
P.pygmaeus      58 GASGPAEGRVARGSERFRDLVPNYNPDIIFKDEENSGADRLMTERCKERVNALAIAVMNM
C.familiaris    58 GASGPAEGRVARGSERFRDLVPNYNPDIIFKDEENSGADRLMTERCKERVNALAIAVMNM
M.musculus      58 GASGPAEGRVTRGSERFRDLVPNYNPDIIFKDEENSGADRLMTERCKERVNALAIAVMNM
M.eugenii       61 GASGQPEGRVVRGSERFRDLVPNYNPDIIFKDEENSGADRLMTERCKERVNALAIAVMNM

H.sapiens      118 WPGVRLRVTEGWDEDGHHAQDSLHYEGRALDITTSDRDRNKYGLLARLAVEAGFDWVYYE
P.pygmaeus     118 WPGVRLRVTEGWDEDGHHAQDSLHYEGRALDITTSDRDRNKYGLLARLAVEAGFDWVYYE
C.familiaris   118 WPGVRLRVTEGWDEDGHHAQDSLHYEGRALDITTSDRDRNKYGLLARLAVEAGFDWVYYE
M.musculus     118 WPGVRLRVTEGWDEDGHHAQDSLHYEGRALDITTSDRDRNKYGLLARLAVEAGFDWVYYE
M.eugenii      121 WPGVRLRVTEGWDEDGHHAQDSLHYEGRALDITTSDRDRNKYGLLARLAVEAGFDWVHYE

                                       ***
H.sapiens      178 SRNHVHVSVKADNSLAVRAGGCFPGNATVRLWSGERKGLRELHRGDWVLAADASGRVVPT
P.pygmaeus     178 SRNHVHVSVKADNSLAVRAGGCFPGNATVRLWSGERKGLRELHRGDWVLAADASGRVVPT
C.familiaris   178 SRNHVHVSVKAG----TVGGGCFRETEAAQLW-GDARGLRELHR-AWVLAADAAGRVVPT
M.musculus     178 SRNHIHVSVKADNSLAVRAGGCFPGNATVRLRSGERKGLRELHRGDWVLAADAAGRVVPT
M.eugenii      181 SRNHVHVSVKADNSLAVRAGGCFPGNATVRLQSGERKGLRELHHGDWVLAADAAGRVVPT

H.sapiens      238 PVLLFLDRDLQRRASFVAVETEWPPRKLLLTPWHLVFAARGPAPAPGDFAPVFARRLRAG
P.pygmaeus     238 PVLLFLDRDLQRRASFVAVETEWPPRKLLLTPWHLVFAARGPAPAPGDFAPVFARRLRAG
C.familiaris   232 PVLLFLDRDLQRRASFVAVETERPPRKLLLTPWHLVFAARGPAPAPGDFAPVFARRLRAG
M.musculus     238 PVLLFLDRDLQRRASFVAVETERPPRKLLLTPWHLVFAARGPAPAPGDFAPVFARRLRAG
M.eugenii      241 PVLLFLDRDLQRRASFVAVETERPPRKLLLTPRHLVFAARGPAPAPADFEPVFARRLRAG

H.sapiens      298 DSVLAPGGDALRPARVARVAREEAVGVFAPLTAHGTLLVNDVLASCYAVLESHQWAHRAF
P.pygmaeus     298 DSVLAPGGDALRPARVARVAREEAVGVFAPLTAHGTLLVNDVLASCYAVLESHQWAHRAF
C.familiaris   292 DSVLAPGGDALRPARVARVAREEAVGVFAPLTAHGTLLVNDVLASCYAVLESHQWAHRAF
M.musculus     298 DSVLAPGGDALQPARVARVAREEAVGVFAPLTAHGTLLVNDVLASCYAVLESHQWAHRAF
M.eugenii      301 DSVLAPGGDALRPARVARVAREEAVGVFAPLTAHGTLLVNDVLASCYAVLESHQRAHRAF

H.sapiens      358 APLRLLHALGALLPGGAVQPTGMHWYSRLLYRLAEELLG
P.pygmaeus     358 APLRLLHALGALLPGGAVQPTGMHWYSRLLYRLAEELLG
C.familiaris   352 APLRLLHALGALLPGGAVQPTGMHWYSRFLYRLAEELLG
M.musculus     358 APLRLLHALGALLPGGAVQPTGMHWYSRLLYRLAEELMG
M.eugenii      361 APLRLLHALGALLPGGTTQSIGMQWYSRLLYRLAEELLG
```
