# Supplementary material for: Desert hedgehog is a mammal-specific gene expressed during testicular and ovarian development in a marsupial
Source: BMC Dev Biol. 2011 Dec 1;11:72. doi: 10.1186/1471-213X-11-72 (PMC3293750; doi:10.1186/1471-213X-11-72)
Supplement: Additional file 5 — Alignment of tammar Ptch1 protein sequence with four eutherian mammals. Dark shading indicates agreement in at least 60% of the sequences, light shading indicates amino acid similarity to consensus. Double dashed areas represent putative trans-membrane binding domains, with species (Ptch1 unless indicated) showing highest sequence identity indicated in parentheses. Any conserved domains are mentioned above the relative sequence. Glycosylation sites are denoted with a cross. [file 1471-213X-11-72-S5.PDF]

```
P.pygmaeus     1 MASAGNAAEPQDRGGGGSGCIGAPGRPAGGG---RRTRTGGLRRAAAPDRDYLHRPSYCD
H.sapiens      1 MASAGNAAEPQDRGGGGSGCIGAPGRPAGGG---RRRRTGGLRRAAAPDRDYLHRPSYCD
B.taurus       1 MASAGNAAELQNRGGGGS-CSGAPGRPAGGG---RRRRTGGLRRNAVPDWDYLHRPSYCD
M.musculus     1 MASAGNAA-------------GALGRQAGGG---RRRRTGGPHR-AAPDRDYLHRPSYCD
M.eugenii      1 MASAVNIAEPESGGGGGSGGCGRDPSLPGGNGSRRRRRTGGSRRAGAPDLEYLQRPSYCD
                                              (Homo sapiens)
                                    Putative sterol transport fam.
                                         ========100%=======
P.pygmaeus    58 AAFALE-ISKGKATGRKAPLWLRAKFQRLLFKLGCYIQKNCGKFLVVGLLIFGAFAVGLK
H.sapiens     58 AAFALEQISKGKATGRKAPLWLRAKFQRLLFKLGCYIQKNCGKFLVVGLLIFGAFAVGLK
B.taurus      57 AAFALEQISKGKATGRKAPLWLRAKFQRLLFKLGCYIQKNCGKFLVVGLLIFGAFAVGLK
M.musculus    44 AAFALEQISKGKATGRKAPLWLRAKFQRLLFKLGCYIQKNCGKFLVVGLLIFGAFAVGLK
M.eugenii     61 AAFALEQISKGKATGRKAPLWLRAKFQRLLFKLGCYIQKNCGKFLVVGLLIFGAFAVGLK

P.pygmaeus   117 AANLETNVEELWVEVGGRVSRELNYTRQKIGEEAMFNPQLMIQTPKEEGANILTTEALLQ
H.sapiens    118 AANLETNVEELWVEVGGRVSRELNYTRQKIGEEAMFNPQLMIQTPKEEGANVLTTEALLQ
B.taurus     117 AANLETNVEELWVEVGGRVSRELNYTRQKIGEEAMFNPQLMIQTPKEEGANVLTTEALRQ
M.musculus   104 AANLETNVEELWVEVGGRVSRELNYTRQKIGEEAMFNPQLMIQTPKEEGANVLTTEALLQ
M.eugenii    121 AANLETNVEELWVEVGGRVSRELNYTRQKIGEEAMFNPQLMIQTPREDGANVLTVEALKQ

P.pygmaeus   177 HLDSALQASRVHVYMYNRQWKLEHLCYKSGELITETGYMDQIIEYLYPCLIITPLDCFWE
H.sapiens    178 HLDSALQASRVHVYMYNRQWKLEHLCYKSGELITETGYMDQIIEYLYPCLIITPLDCFWE
B.taurus     177 HLDSALQASRVHVYMYNRQWKLEHLCYKSGELITETGYMDQIIEYLYPCLIITPLDCFWE
M.musculus   164 HLDSALQASRVHVYMYNRQWKLEHLCYKSGELITETGYMDQIIEYLYPCLIITPLDCFWE
M.eugenii    181 HLDSALQASRVHVYMYNRQWKLEHLCYKSGELITETGYMDQIIEYLYPCLIITPLDCFWE

P.pygmaeus   237 GAKLQSGTAYLLGKPPLRWTNFDPLEFLEELKKINYQVDSWEEMLNKAEVGHGYMDRPCL
H.sapiens    238 GAKLQSGTAYLLGKPPLRWTNFDPLEFLEELKKINYQVDSWEEMLNKAEVGHGYMDRPCL
B.taurus     237 GAKLQSGTAYLLGKPPLQWTNFDPLEFLEELKKINYQVDSWEEMLNKAEVGHGYMDRPCL
M.musculus   224 GAKLQSGTAYLLGKPPLRWTNFDPLEFLEELKKINYQVDSWEEMLNKAEVGHGYMDRPCL
M.eugenii    241 GAKLQSGTAYLLGKPPLQWTNFDPLEFLEELKKINYQVDSWEEMLNKAEVGHGYMDRPCL

                                                   +
                                                   +
P.pygmaeus   297 NPADPDCPATAPNKNSTKPLDMALVLNGGCHGLSRKYMHWQEELIVGGTVKNSTGKLISA
H.sapiens    298 NPADPDCPATAPNKNSTKPLDMALVLNGGCHGLSRKYMHWQEELIVGGTVKNSTGKLVSA
B.taurus     297 NPADPDCPATAPNKNATKPLDMALVLNGGCHGLSRKYMHWQEELIVGGTVKNSTGKLVSA
M.musculus   284 NPADPDCPATAPNKNSTKPLDVALVLNGGCQGLSRKYMHWQEELIVGGTVKNATGKLVSA
M.eugenii    301 SPADPDCPVTAPNKNSTRPLDVALVLNGGCHGLSRKYMHWQEELIVGGTVKNSTGKLVSA

P.pygmaeus   357 HALQTMFQLMTPKQMYEHFKGYEYVSHINWNEDKAAAILEAWQRTYVEVVHQSVAQNSTQ
H.sapiens    358 HALQTMFQLMTPKQMYEHFKGYEYVSHINWNEDKAAAILEAWQRTYVEVVHQSVAQNSTQ
B.taurus     357 HALQTMFQLMTPKQMYEHFKGYEYVSHINWNEDKAAAILEAWQRTYVEVVHQSVAQNSTQ
M.musculus   344 HALQTMFQLMTPKQMYEHFRGYDYVSHINWNEDRAAAILEAWQRTYVEVVHQSVAPNSTQ
M.eugenii    361 QALQTMFQLMTPKQMYEHFKGYEYVSHINWNEDKAAAILEAWQRMYVEVVHQSVAQNSTQ
```

```
                  (Homo sapiens)                       (Danio rerio)
               Putative sterol transport fam.     Putative sterol transport fam.
                  =======100%=========               ==========100%========
P.pygmaeus   417 KVLSFTTTTLDDILKSFSDVSVIRVASGYLLMLAYACLTMLRWDCSKSQGAVGLAGVLLV
H.sapiens    418 KVLSFTTTTLDDILKSFSDVSVIRVASGYLLMLAYACLTMLRWDCSKSQGAVGLAGVLLV
B.taurus     417 KVLSFTTTTLDDILKSFSDVSVIRVASGYLLMLAYACLTMLRWDCSRSQGAVGLAGVLLV
M.musculus   404 KVLPFTTTTLDDILKSFSDVSVIRVASGYLLMLAYACLTMLRWDCSKSQGAVGLAGVLLV
M.eugenii    421 KVLSFTTTTLDDILKSFSDVSVIRVASGYLLMLAYACLTMLRWDCAKSQGAVGLAGVLLV
                         (Homo sapiens)
                  Putative sterol transport fam.
                      =======100%========                       =======
P.pygmaeus   477 ALSVAAGLGLCSLIGISFNAATTQVLPFLALGVGVDDVFLLAHAFSETGQNKRIPFEDRT
H.sapiens    478 ALSVAAGLGLCSLIGISFNAATTQVLPFLALGVGVDDVFLLAHAFSETGQNKRIPFEDRT
B.taurus     477 ALSVAAGLGLCSLIGISFNAATTQVLPFLALGVGVDDVFLLAHAFSETGQNKRIPFEDRT
M.musculus   464 ALSVAAGLGLCSLIGISFNAATTQVLPFLALGVGVDDVFLLAHAFSETGQNKRIPFEDRT
M.eugenii    481 ALSVAAGLGLCSLIGISFNAATTQVLPFLALGVGVDDVFPLAHAFSETGQNKRIPFEDRT
                (Homo sapiens)          (Callorhinchus milii)
                    Putative sterol transport fam. (BOTH)
                 ===100%========        ========100%=========
P.pygmaeus   537 GECLKRTGASVALTSISNVTAFFMAALIPIPALRAFSLQAAVVVVFNFAMVLLIFPAILS
H.sapiens    538 GECLKRTGASVALTSISNVTAFFMAALIPIPALRAFSLQAAVVVVFNFAMVLLIFPAILS
B.taurus     537 GECLKRTGASVALTSISNVTAFFMAALIPIPALRAFSLQAAVVVVFNFAMVLLIFPAILS
M.musculus   524 GECLKRTGASVALTSISNVTAFFMAALIPIPALRAFSLQAAVVVVFNFAMVLLIFPAILS
M.eugenii    541 GECLKRTGASVALTSISNVTAFFMAALIPIPALRAFSLQAAIVVVFNFAMVLLIFPAILS

P.pygmaeus   597 MDLYRREDRRLDIFCCFTSPCVSRVIQVEPQAYTDTHDNTRYSPPPPYSSHSFAHETQIT
H.sapiens    598 MDLYRREDRRLDIFCCFTSPCVSRVIQVEPQAYTDTHDNTRYSPPPPYSSHSFAHETQIT
B.taurus     597 MDLYRREDRRLDIFCCFTSPCVSRVIQVEPQAYTETHDNTRYSPPPPYSSHGFAHETQIT
M.musculus   584 MDLYRREDRRLDIFCCFTSPCVSRVIQVEPQAYTEPHSNTRYSPPPPYTSHSFAHETHIT
M.eugenii    601 MGLYRREDRRLDIFCCFTSPCVSRVIQVEPQAYTDTNDNTRYSPPPPYSSHSFAHETQIT

P.pygmaeus   657 MQSTVQLRTEYDPHTHVYYTTAEPRSEISVQPVTVTQDTLSCQSPESTSSTRDLLSQFSD
H.sapiens    658 MQSTVQLRTEYDPHTHVYYTTAEPRSEISVQPVTVTQDTLSCQSPESTSSTRDLLSQFSD
B.taurus     657 MQSTVQLRTEYDPHTHVYYTTAEPRSEISVQPVTMAQDTLSCQSPESTSSTRDLLSQFSD
M.musculus   644 MQSTVQLRTEYDPHTHVYYTTAEPRSEISVQPVTVTQDNLSCQSPESTSSTRDLLSQFSD
M.eugenii    661 MQSTVQLRTEYDPHTQVYYTTAEPRSEISVQPVTMTQDNLSCHSPESTSSTRDLLSQFSD
                                  (Homo sapiens)
                          Putative sterol transport fam.
                               =======100%========
P.pygmaeus   717 SSLHCLEPPCTKWTLSSFAEKHYAPFLLKPKAKVVVIFLFLGLLGVSLYGTTRVRDGLDL
H.sapiens    718 SSLHCLEPPCTKWTLSSFAEKHYAPFLLKPKAKVVVIFLFLGLLGVSLYGTTRVRDGLDL
B.taurus     717 SSLHCLEPPCTRWTLSSFAEKHYAPFLLKPKAKVVVIFLFLGLLGVSLYGTTRVRDGLDL
M.musculus   704 SSLHCLEPPCTKWTLSSFAEKHYAPFLLKPKAKVVVILLFLGLLGVSLYGTTRVRDGLDL
M.eugenii    721 SNLHCLEPPCTKWTLSSFAEKHYAPFLLKPKAKVVVILLFLGLLGVSLYGTTRVRDGLDL

P.pygmaeus   777 TDIVPRETREYDFIAAQFKYFSFYNMYIVTQKADYPNIQHLLYDLHRSFSNVKYVMLEEN
H.sapiens    778 TDIVPRETREYDFIAAQFKYFSFYNMYIVTQKADYPNIQHLLYDLHRSFSNVKYVMLEEN
B.taurus     777 TDIVPRETREYDFIAAQFKYFSFYNMYIVTQKADYPNIQHLLYDLHKSFSNVKYVMLEEN
M.musculus   764 TDIVPRETREYDFIAAQFKYFSFYNMYIVTQKADYPNIQHLLYDLHKSFSNVKYVMLEEN
M.eugenii    781 TDIVPRETREYDFIAAQFKYFSFYNMYIVTQKADYPHIQHLLYDLHKSFSSVKYVMLEEN

                                       +
                                       +
P.pygmaeus   837 KQLPK-WLHYFRDWLQGLQDAFDSDWETGKIMPNNYKNGSDDGVLAYKLLVQTGSRDKPI
H.sapiens    838 KQLPKMWLHYFRDWLQGLQDAFDSDWETGKIMPNNYKNGSDDGVLAYKLLVQTGSRDKPI
B.taurus     837 KQLPKMWLHYFRDWLQGLQDAFDSDWETGKIMPNNYKNGSDDGVLAYKLLVQTGSRDKPI
```

```
M.musculus  824 KQLPQMWLHYFRDWLQGLQDAFDSDWETGRIMPNNYKNGSDDGVLAYKLLVQTGSRDKPI
M.eugenii   841 KELPKMWLHYFRDWLQGLQDAFDSDWESGKIMQNNYKNGSDDGVLAYKLLVQTGSRDKPI


P.pygmaeus  896 DISQLTKQRLVDADGIINPSAFYIYLTAWVSNDPVAYAASQANIRPHRPEWVHDKADYMP
H.sapiens   898 DISQLTKQRLVDADGIINPSAFYIYLTAWVSNDPVAYAASQANIRPHRPEWVHDKADYMP
B.taurus    897 DISQLTKRRLVDADGIINPSAFYIYLTAWVSNDPVAYAASQANIRPHRPEWVHDKADYMP
M.musculus  884 DISQLTKQRLVDADGIINPSAFYIYLTAWVSNDPVAYAASQANIRPHRPEWVHDKADYMP
M.eugenii   901 DISQLTKQRLVDADGIINPSAFYIYLTAWVSNDPVAYAASQANIRPHRPEWVHDKADYMP

                                                                      ======
P.pygmaeus  956 ETRLRIPAAEPIEYAQFPFYLNGLRDTSDFVEAIEKVRTICSNYTSLGLSSYPNGYPFLF
H.sapiens   958 ETRLRIPAAEPIEYAQFPFYLNGLRDTSDFVEAIEKVRTICSNYTSLGLSSYPNGYPFLF
B.taurus    957 ETRLRIPAAEPIEYAQFPFYLNGLRDTSDFVEAIEKVRTICNNYTSLGLSSYPNGYPFLF
M.musculus  944 ETRLRIPAAEPIEYAQFPFYLNGLRDTSDFVEAIEKVRVICNNYTSLGLSSYPNGYPFLF
M.eugenii   961 DTRLRIPPAEPIEYAQFPFYLNGLRDTSDFVEAIEKVRTICNNYTSLGVSSYPNGYPFLF
             (Polyodon spathula)       (Homo sapiens)        (Homo sapiens)
                       Putative sterol transport family (All three)
                ==100%=========     ========100%=========    ========100%===
P.pygmaeus 1016 WEQYIGLRHWLLLFISVVLACTFLVCAVFLLNPWTAGIIVVVLALMTVELFGMMGLIGIK
H.sapiens  1018 WEQYIGLRHWLLLFISVVLACTFLVCAVFLLNPWTAGIIVMVLALMTVELFGMMGLIGIK
B.taurus   1017 WEQYIGLRHWLLLSISVVLACTFLVCAVFLLNPWTAGIIVTVLALMTVELFGMMGLIGIK
M.musculus 1004 WEQYISLRHWLLLSISVVLACTFLVCAVFLLNPWTAGIIVMVLALMTVELFGMMGLIGIK
M.eugenii  1021 WEQYIGLRHWLLLSISVVLACTFLVCAVFLLNPWTAGIIVMVLALMTVELFGMMGLIGIK
                                                   (Homo sapiens)
                                           Putative sterol transport fam.
                ======                       ========100%========
P.pygmaeus 1076 LSAVPVVILIASVGIGVEFTVHVALAFLTAIGDKNRRAVLALEHMFAPVLDGAVSTLLGV
H.sapiens  1078 LSAVPVVILIASVGIGVEFTVHVALAFLTAIGDKNRRAVLALEHMFAPVLDGAVSTLLGV
B.taurus   1077 LSAVPVVILIASVGIGVEFTVHVALAFLTAIGDKNRRAVLALEHMFAPVLDGAVSTLLGV
M.musculus 1064 LSAVPVVILIASVGIGVEFTVHVALAFLTAIGDKNHRAMLALEHMFAPVLDGAVSTLLGV
M.eugenii  1081 LSAVPVVILIASVGIGVEFTVHVALAFLTAIGDKNRRAVLALEHMFAPVLDGAVSTLLGV
                      (Homo sapiens)
                Putative sterol transport fam.
                   =========100%=========
P.pygmaeus 1136 LMLAGSEFDFIVRYFFAVLAILTILGVLNGLVLLPVLLSFFGPYPEVSPANGLNRLPTPS
H.sapiens  1138 LMLAGSEFDFIVRYFFAVLAILTILGVLNGLVLLPVLLSFFGPYPEVSPANGLNRLPTPS
B.taurus   1137 LMLAGSEFDFIVRYFFAVLAILTILGVLNGLVLLPVLLSFFGPYPEVSPANGLNRLPTPS
M.musculus 1124 LMLAGSEFDFIVRYFFAVLAILTVLGVLNGLVLLPVLLSFFGPCPEVSPANGLNRLPTPS
M.eugenii  1141 LMLAGSEFDFIVRYFFAVLAILTILGVLNGLVLLPVLLSFFGPYPEVTPANGLNRLPTPS


P.pygmaeus 1196 PEPPPSVVRFAMPPGHMHSGSDSSDSEYSSQTTVSGLSEELRHYEAQQAAGGPAHQVIVE
H.sapiens  1198 PEPPPSVVRFAMPPGHTHSGSDSSDSEYSSQTTVSGLSEELRHYEAQQGAGGPAHQVIVE
B.taurus   1197 PEPPPSVVRFAVPAGHTNNGSDSSDSEYSSQTTVSGISEELRHYEAQQGAGGPAHQVIVE
M.musculus 1184 PEPPPSVVRFAVPPGHTNNGSDSSDSEYSSQTTVSGISEELRQYEAQQGAGGPAHQVIVE
M.eugenii  1201 PEPPPSTVRFAVPPRHTNNGSDSSDSEYSSQTTVSGISEELYQYETQQSSCAPAHQVIVE


P.pygmaeus 1256 ATENPVFAHSTVVHPESRHHPPSNPRQQ----------------PRRDPPREGLWPPPYR
H.sapiens  1258 ATENPVFAHSTVVHPESRHHPPSNPRQQPHLDSGSLPPGRQGQQPRRDPPREGLWPPPYR
B.taurus   1257 ATENPVFARSTVVHPEPRHHPPSNPRQQSHLDSRTLPPGQPGQQPRRETPRECLRAPPYK
M.musculus 1244 ATENPVFARSTVVHPDSRHQPPLTPRQQPHLDSGSLSPGRQGQQPRRDPPREGLRPPPYR
M.eugenii  1261 ATENPVFARSTVVQPEPRHHHPSSPRQQLHLDAGPHQPGHQGQQPQRDS-REGLRPPPYR


P.pygmaeus 1300 PRRDAFEISTEGHSGPSNRDRWGPRGARSHNPR--NPASTAMGSSMPGYCQPITTVTASA
H.sapiens  1318 PRRDAFEISTEGHSGPSNRARWGPRGARSHNPR--NPASTAMGSSVPGYCQPITTVTASA
B.taurus   1317 PRRDAFEISTEGHSGPSNRDRTGPRGARSHNPRHHNPAFTAMGSSMPSYCQPITTVTASA
```

```
M.musculus 1304 PRRDAFEISTEGHSGPSNRDRSGPRGARSHNPR--NPTSTAMGSSVPSYCQPITTVTASA
M.eugenii  1320 PRRNAFEISTDGHSGPSNRDRVGHRGARFHNPR--NPAFTAMGTSVPGYCQPITTVTASA
```

```
P.pygmaeus 1358 SVTVAVHPPPVPGPG--RNPRGGLCPG---YPETDHGLFEDPHVPFHVRRERRDSKVEVI
H.sapiens  1376 SVTVAVHPPPVPGPG--RNPRGGLCPG---YPETDHGLFEDPHVPFHVRCERRDSKVEVI
B.taurus   1377 SVTVAVHPPPAPGPGPSRNPRSGLCPGYEDYPETDHGLFEDPHVPFNVRCERREPKVEVI
M.musculus 1362 SVTVAVHPP--PGPG--RNPRGGPCPGYESYPETDHGVFEDPHVPFHVRCERRDSKVEVI
M.eugenii  1378 SVTVAVHPP--PMHG--RNPWGGSCPSYEGYHETDHGVFEDPHVPFNVRCERRNSKIEVI
```

```
P.pygmaeus 1413 ELQDVECEERPRGSNSN
H.sapiens  1431 ELQDVECEERPRGSSSN
B.taurus   1437 ELQDVECEERRHGSSSN
M.musculus 1418 ELQDVECEERPWGSSSN
M.eugenii  1434 ELQDVECEERTKGNSSN
```
