# Supplementary material for: Desert hedgehog is a mammal-specific gene expressed during testicular and ovarian development in a marsupial
Source: BMC Dev Biol. 2011 Dec 1;11:72. doi: 10.1186/1471-213X-11-72 (PMC3293750; doi:10.1186/1471-213X-11-72)

A.

```
                                                                                      ===
C.famil.     1 MARPPPLQELPPGYTPPARATSPQIPAGSLKAPLWLRAYFQGLLFSLGCGIQRHCGKVLF
B.taurus     1 MARPPPLGELPPGYTPPGRPATPQILAGSLKAPLWLRAYFQGLLFSLGCGIQRHCGKVLF
H.sapiens    1 MTRSPPLRELPPSYTPPARTAAPQILAGSLKAPLWLRAYFQGLLFSLGCGIQRHCGKVLF
M.musculus   1 MVRPLSLGELPPSYTPPARSSAPHILAGSLQAPLWLRAYFQGLLFSLGCRIQKHCGKVLF
M.eugenii    1 --------------MAPGELLTSGPRAPSAGAPLWLRAHVGGLLFGLGCAIQQHCGKVLF

               (Homo sapiens)
               ===95%==========
C.famil.    61 LGLLAFGALALGLRVAIVETDLEQLWVEVGSRVSQELHYTKEKLGEEAAYTSQMLIQTPR
B.taurus    61 LGLLAFGALALGLRVAIIETDLEQLWVEVGSRVSQELEYTKEKLGEEAAYTSQMLIQTPR
H.sapiens   61 LGLLAFGALALGLRMAIIETNLEQLWVEVGSRVSQELHYTKEKLGEEAAYTSQMLIQTAR
M.musculus  61 LGLVAFGALALGLRVAVIETDLEQLWVEVGSRVSQELHYTKEKLGEEAAYTSQMLIQTAH
M.eugenii   47 VGLLAFGALALGLRGAAVETDLERLWVEVGSRVSQELRYTKEKLGEEAVYTSQTLIQTAH

C.famil.   121 QEGENVLTPEALGLHLQAALTASKVQVSLYGKSWDLNKICYKSGIPLIENGMIERMIEKL
B.taurus   121 QEGENVLTPEALDLHLQAALTASKVQVSLYGKSWDLNKICYKSGVPLIENGMIERMIEKL
H.sapiens  121 QEGENILTPEALGLHLQAALTASKVQVSLYGKSWDLNKICYKSGVPLIENGMIERMIEKL
M.musculus121 QEGGNVLTPEALDLHLQAALTASKVQVSLYGKSWDLNKICYKSGVPLIENGMIERMIEKL
M.eugenii  107 GASESVLTPEALGLHLQAALAASKVQVSLYGKSWDLNKICYKAGVPIIENGMIERMIEKL

C.famil.   181 FPCVILTPLDCFWEGAKLQGGSAYLPGRPDIQWTNLDPEQLLEELGPFASLEGFRELLDK
B.taurus   181 FPCVILTPLDCFWEGAKLQGGSAYLPGRPDIQWTNLDPEQLLAELGPFASLEGFRELLDK
H.sapiens  181 FPCVILTPLDCFWEGAKLQGGSAYLPGRPDIQWTNLDPEQLLEELGPFASLEGFRELLDK
M.musculus181 FPCVILTPLDCFWEGAKLQGGSAYLPGRPDIQWTNLDPQQLLEELGPFASLEGFRELLDK
M.eugenii  167 FPCVILTPLDCFWEGAKLQGGSAYLPGRPDIQWTNLDPEQLLEELGPFASLEGFRELLNK

C.famil.   241 AQVGQAYVGRPCLHPDDLHCPPSAPNHHSKQAPNVAQELSGGCHGFSHKFMHWQEELLLG
B.taurus   241 AQVGQAYVGRPCLHPDDLHCPPSAPNHHSRQAPNVAQELSGGCHGFSHKFMHWQEELLLG
H.sapiens  241 AQVGQAYVGRPCLHPDDLHCPPSAPNHHSRQAPNVAHELSGGCHGFSHKFMHWQEELLLG
M.musculus241 AQVGQAYVGRPCLDPDDPHCPPSAPNRHSRQAPNVAQELSGGCHGFSHKFMHWQEELLLG
M.eugenii  227 AQVGQAYVGRPCLHPDDPHCPASAPNHHSRQVPDIARELSGGCHGFSRKFMHWQEELLLG

C.famil.   301 GMARDPQGQLLRAEALQSTFLLMSPRQLYEHFRGDYQTHDIGWSEEQAGTVLQAWQRRFV
B.taurus   301 GMARDPQGQLLRAEALQSTFLLMSPRQLYEHFRGDYQTHDIGWSEEQAGTVLQAWQRRFV
H.sapiens  301 GMARDPQGELLRAEALQSTFLLMSPRQLYEHFRGDYQTHDIGWSEEQASTVLQAWQRRFV
M.musculus301 GTARDLQGQLLRAEALQSTFLLMSPRQLYEHFRGDYQTHDIGWSEEQASMVLQAWQRRFV
M.eugenii  287 SPVRSPQGRLLSAEALQSTFLLMSPRQLYDHYRGDYETHDISWSEAQAGAVLQAWQRRFV
                                                     (Leucoraja erinacea)
                                              Putative sterol transport fam.
                                                     ========100%========
C.famil.   361 QLAQEALPQNSSQQIHAFSSTTLDDILHAFSEVSAARVVGGYLLMLAYACVTMLRWDCAQ
B.taurus   361 QLAQEALPENASQQIHAFSSTTLDDILHAFSEVSAARVVGGYLLMLAYACVTMLRWDCAQ
H.sapiens  361 QLAQEALPENASQQIHAFSSTTLDDILHAFSEVSAARVVGGYLLMLAYACVTMLRWDCAQ
M.musculus361 QLAQEALPANASQQIHAFSSTTLDDILRAFSEVSTTRVVGGYLLMLAYACVTMLRWDCAQ
M.eugenii  347 ELAQQSVPQNASQQIHAFSATTLDDLLRSFSDISAVRVAGGYLLMLAYACVTMLRWDCSK

                      (Danio rerio)             (Scyliorhinus canicula)
                  =========95%==========        ========100%=======
C.famil.   421 SQGAVGLAGVLLVALAVASGLGLCALLGIAFNAATTQVLPFLALGIGVDDIFLLAHAFTE
B.taurus   421 SQGAVGLAGVLLVALAVASGLGLCALLGIAFNAATTQVLPFLALGIGVDDIFLLAHAFTE
H.sapiens  421 SQGSVGLAGVLLVALAVASGLGLCALLGITFNAATTQVLPFLALGIGVDDVFLLAHAFTE
M.musculus421 SQGAVGLAGVLLVALAVASGLGLCALLGITFNAATTQVLPFLALGIGVDDIFLLAHAFTK
```

```
M.eugenii 407 SQGAVGLAGVLLVALSVASGLGLCSLLGMTFNAATTQVLPFLALGIGVDDMFLLAHAFTE
                                        (Mus musculus)
                               Putative sterol transport fam.
                                  =========100%==========        =========
C.famil.  481 APPGTPLQERTGECLQRTGTSVALTSISHMVAFFMAALVPIPALRAFSLQAAIVVGCNFA
B.taurus  481 APPGSPLQERTGECLRRTGTSVTLTSINNMVAFFMAALVPIPALRAFSLQAAIVVGCNFA
H.sapiens 481 ALPGTPLQERMGECLQRTGTSVVLTSINNMAAFLMAALVPIPALRAFSLQAAIVVGCTFV
M.musculus481 APPDTPLPERMGECLRSTGTSVALTSVNNMVAFFMAALVPIPALRAFSLQAAIVVGCNFA
M.eugenii 467 APSGIPLQERTGECLQRMGTSVALTSVNNLVAFFMAALVPIPALRAFSLQAAVVVSCNFT
         (Mus musculus)
  Putative sterol transport fam.
              =90%========
C.famil.  541 AVMLVFPAVLSLDLHRRHCQRLDVLCCFSSPCSARVIQILPQELGDGTVPVGIAHLTATV
B.taurus  541 AVMLVFPAVLSLDLRRRHCRRLDVLCCFSSPCSARVIQILPQELGNGTVPVGVAHLTATV
H.sapiens 541 AVMLVFPAILSLDLRRRHCQRLDVLCCFSSPCSAQVIQILPQELGDGTVPVGIAHLTATV
M.musculus541 AVMLVFPAILSLDLRRRHRQRLDVLCCFSSPCSAQVIQMLPQELGDRAVPVGIAHLTATV
M.eugenii 527 AVLLIFPAILSLDLHRRHRQRLDVLCCFSSPCSSRVIQIQPQELGEVQMPV--THLTATV

C.famil.  601 QAFAHCEAGSQHVVTILPPRARLVPPPSDPLGSELFSPGGSTRDLLGQEEGTRQKATCSS
B.taurus  601 QAFAHCEASSQHVVTILPPQAQLVPPPSDPLGSELFSPGGSTRDLLGQEEGTGQKAACKS
H.sapiens 601 QAFTHCEASSQHVVTILPPQAHLVPPPSDPLGSELFSPGGSTRDLLGQEEETRQKAACKS
M.musculus601 QAFTHCEASSQHVVTILPPQAHLLSPASDPLGSELYSPGGSTRDLLSQEEGTGPQAACRP
M.eugenii 585 QAFARCDTAGQHVVTILPPTTHLTPLPTEPLGSQLFGPMGSTRDLLGQVAGTGRGQVCRP

                                          (Homo sapiens)
                                       ========90%========
C.famil.  661 LPCARWNLAHFARSQFAPLLLQSHSKAIVLVLFGALLGLSLYGATLVQDGLALTDVVPRG
B.taurus  661 LPCARWNLAHFARSQFAPLLLQSHTKAVVLVLFGALLGLSLYGATLVQDGLALTDVVPRG
H.sapiens 661 LPCARWNLAHFARYQFAPLLLQSHAKAIVLVLFGALLGLSLYGATLVQDGLALTDVVPRG
M.musculus661 LLCAHWTLAHFARYQFAPLLLQTRAKALVLLFFGALLGLSLYGATLVQDGLALTDVVPRG
M.eugenii 645 LPCARWNLSRFARCQYAPLLLQPRTKGLVLVLFGALLGLSLYGATWVQDGLTLTDVVPRG

C.famil.  721 TKEHAFLSAQLRYFSLYEVALVTQGGFDYAHSQRALFDLHQRFSSLKAVLPTPATQAPRT
B.taurus  721 TKEHAFLSAQLRYFSLYEVALVTQGGFDYAHSQRALFDLHQRFSSLKAVLPPPATQAPRT
H.sapiens 721 TKEHAFLSAQLRYFSLYEVALVTQGGFDYAHSQRALFDLHQRFSSLKAVLPPPATQAPRT
M.musculus721 TKEHAFLSAQLRYFSLYEVALVTQGGFDYAHSQRALFDLHQRFSSLKAVLPPPATQAPRT
M.eugenii 705 TKEYDFLAAQIKYFSLYEVALVTQGGFDYAHSQQALLDLHSRFSALKSVLAP---QPPRS

C.famil.  781 WLHYYRNWLLGIQAAFDQDWASGRISRHSCRNGSEDGALAYKLLLQTGDAQEPLDFSQLT
B.taurus  781 WLHYYRSWLQGIQAAFDQDWASGRITRHSYRNGSEDGALAYKLLVQTGDAQEPLDFSQLT
H.sapiens 781 WLHYYRNWLQGIQAAFDQDWASGRITRHSYRNGSEDGALAYKLLIQTGDAQEPLDFSQLT
M.musculus781 WLHYYRSWLQGIQAAFDQDWASGRITCHSYRNGSEDGALAYKLLIQTGNAQEPLDFSQLT
M.eugenii 762 WLHRYSAWLQGIQAAFDQDWEAGRITRHSCRNGSEDGALAYRLLIQTGDAKEPLDYSQLD

C.famil.  841 TRKLVDKEGLIAPELFYVGLTMWVSSDPLGLAASQANFYPPPPEWLHDKYDTTGENLRIP
B.taurus  841 TKKLVDKEGLIPPELFYMGLTVWVSSDPLGLAASQANFYPPPPEWLHDKYDTTGENLRIP
H.sapiens 841 TRKLVDREGLIPPELFYMGLTVWVSSDPLGLAASQANFYPPPPEWLHDKYDTTGENLRIP
M.musculus841 TRKLVDKEGLIPPELFYMGLTVWVSSDPLGLAASQANFYPPPPEWLHDKYDTTGENLRIP
M.eugenii 822 KRKLVNADGLILPELFYVGLTVWVSRDPLGLAASQANFYPPPPEWLHDKYDSPGESLHIP

C.famil.  901 AAQPLEFAQFPFLLRGLQKTADFVEAIEGARAACAEAGQAGVRAYPSGSPFLFWEQYLGL
B.taurus  901 AAQPLEFAQFPFLLRGLQKTADFVEAIEGARAACAEASQAGVHAYPSGSPFLFWEQYLGL
H.sapiens 901 PAQPLEFAQFPFLLRGLQKTADFVEAIEGARAACAEAGQAGVHAYPSGSPFLFWEQYLGL
M.musculus901 AAQPLEFAQFPFLLHGLQKTADFVEAIEGARAACTEAGQAGVHAYPSGSPFLFWEQYLGL
```

```
                                             (Homo sapiens)
M.eugenii 882 AAPPLEFAQFPFLLSGLRQTADFVEAIEGARAACEEAGQAGIRAYPSGSPFLFWEQYLGL
                     (Homo sapiens)     Putative sterol transport fam.
                 =========90%=========    =========100%=========   =======
C.famil.  961 RRYFLLAICILLVCTFLVCALLLLNPWTAGLIVLVLAMMTVELFGIMGFLGIKLSAIPVV
B.taurus  961 RRCFLLAVCILLLCTFLVCALLLLNPWTAALIVLVLAMMTVELFGIMGFLGIKLSAIPVV
H.sapiens 961 RRCFLLAVCILLVCTFLVCALLLLNPWTAGLIVLVLAMMTVELFGIMGFLGIKLSAIPVV
M.musculus961 RRCFLLAVCILLVCTFLVCALLLLSPWTAGLIVLVLAMMTVELFGIMGFLGIKLSAIPVV
M.eugenii 942 RRCFLLAVCVLLACTFVVCALLLLSPWTAGLIVLVLAMMTVELFGIMGFLGIKLSAIPVV
              (Homo sapiens)                       (Eublepharis macularius)
      Putative sterol transport fam.           Putative sterol transport fam.
              ==95%=========                       =========95%========
Canis    1021 ILVASVGIGVEFTVHVALLRIGSSPCSGTRLKKWGKYKQTKGPEQGTGLVPDLGILSLAS
B.taurus 1021 ILVASIGIGVEFTVHVALGFLTAQ--GSRNLRAARALERTFAPVTDGAISTLLGLLMLAG
H.sapiens1021 ILVASVGIGVEFTVHVALGFLTTQ--GSRNLRAAHALEHTFAPVTDGAISTLLGLLMLAG
Mus      1021 ILVASIGIGVEFTVHVALGFLTSH--GSRNLRAASALEQTFAPVTDGAVSTLLGLLMLAG
M.eugenii1002 ILVASVGIGVEFTAHVALGFLTAT--GSRDVRSAQALEHMFAPVMDGAVSTLLGLLMLAG

                               (Homo sapiens)
                          ===========92%==========
Canis    1081 S-------------CILCTSELLKGTEVLVSLGSVDLCLP--------------------
B.taurus 1079 SNFDFIVRYFFVVLTILTLLGLLHGLVLLPVLLSILGPPP--------------------
H.sapiens1079 SHFDFIVRYFFAALTVLTLLGLLHGLVLLPVLLSILGPPP--------------------
Mus      1079 SNFDFIIRYFFVVLTVLTLLGLLHGLLLLPVLLSILGPPP--------------------
M.eugenii1060 SDFDFIVRYFFVVLTILTGLGLLHGLVLLPVLLSILGPPPQVSLPDGGSHLPHPDPISLP


Canis    1108 --------------------------------------------------QVVQMYKESP
B.taurus 1119 --------------------------------------------------EVVQMYKESA
H.sapiens1119 --------------------------------------------------EVIQMYKESP
Mus      1119 --------------------------------------------------QVVQVYKESP
M.eugenii1120 FSPPHFFLGSSPAFRGPEAGAGDAPSTFILPPTHSHILVEASKDPSFPTITVVQTYKDSP


Canis    1118 E-----VLSPPAPREGGLRWGLPPTLPQSFARVTTSMTVALHPPPLPGAYIHPASDEPT-
B.taurus 1129 E-----VLSPPAPQGGGLRWGVPSTLPQSFARVTTSMTVALHPPPLPGAYIHPASEEPTW
H.sapiens1129 E-----ILSPPAPQGGGLRWGASSSLPQSFARVTTSMTVAIHPPPLPGAYIHPAPDEPPW
Mus      1129 Q-----TLNSAAPQRGGLRWDRPPTLPQSFARVTTSMTVALHPPPLPGAYVHPASEEPT-
M.eugenii1180 PGPGPGLPSLTATAGSEARWG-PHASPGAFTTLTASVTVALHPPPLPGSYVQEVSEEPRH


C.famil.      ------------------------
B.taurus 1184 SPAATPAANGPSNLGPRGLCPATG
H.sapiens1184 SPAATSS----GNLSSRGPGPATG
M.musculus    ------------------------
M.eugenii1239 PLATEPKGSGPCC-----------
```

B.

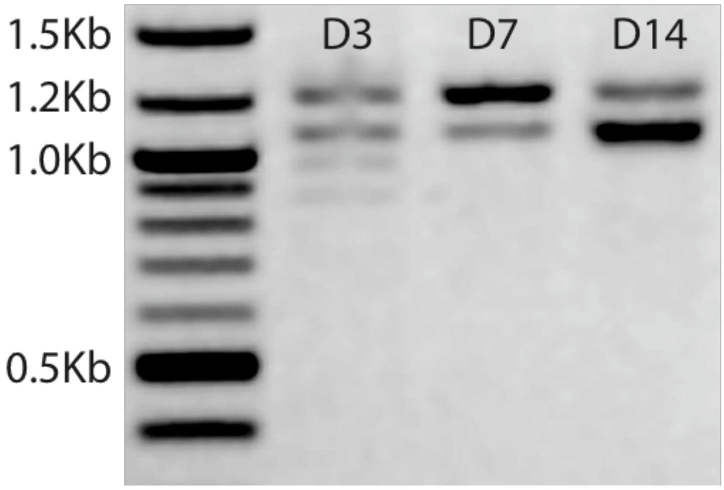

Supplement: Additional file 6 — a. Alignment of tammar Ptch2 protein sequence with four eutherian mammals. Dark shading indicates agreement in at least 60% of the sequences, light shading indicates amino acid similarity to consensus. 70 amino acid stretch maintained in Tammar is italicized. Double dashed areas represent putative trans-membrane binding domains, with species showing highest sequence identity indicated in parentheses. Any conserved domains are mentioned above the relative sequence. b. Alternative splice variants of PTCH2. Primers were designed to span the region corresponding to exons 18-22 of the human PTCH2 gene. RT-PCR was carried out in day 3, 7 and 14 post partum testes. Day 3 PCR produced four bands of ~1.2 Kb, 1.05 Kb, 950 bp and 860 bp. We sequence verified that the 1.2 Kb fragment was the full-length transcript and that the 950 bp transcript was a Δ-21a PTCH2 isoform. The identity of the missing exons in the 1.05 Kb and 860 bp fragments is shown in Additional File 9. These slice variants were developmentally regulated, with the smaller two isoforms not seen in the day 7 or 14 testis and the larger two isoforms appear to change in their relative abundance between stages. [file 1471-213X-11-72-S6.PDF]
